# Digoxin reveals a functional connection between HIV-1 integration preference and T-cell activation

**DOI:** 10.1371/journal.ppat.1006460

**Published:** 2017-07-20

**Authors:** Alexander Zhyvoloup, Anat Melamed, Ian Anderson, Delphine Planas, Chen-Hsuin Lee, Janos Kriston-Vizi, Robin Ketteler, Andy Merritt, Jean-Pierre Routy, Petronela Ancuta, Charles R. M. Bangham, Ariberto Fassati

**Affiliations:** 1 Division of Infection & Immunity, University College London, London, United Kingdom; 2 Department of Medicine, Imperial College, St. Mary's Campus, London, United Kingdom; 3 Department of Microbiology, Infectiology and Immunology, Faculty of Medicine, University of Montreal and the Research Centre of the CHUM, Montreal, Québec, Canada; 4 MRC Laboratory for Molecular Cell Biology, University College London, London, United Kingdom; 5 Centre for Therapeutics Discovery, MRC Technology, Mill Hill, London, United Kingdom; 6 McGill University Health Centre, Glen site, Montreal, Québec, Canada; University of North Carolina at Chapel Hill, UNITED STATES

## Abstract

HIV-1 integrates more frequently into transcribed genes, however the biological significance of HIV-1 integration targeting has remained elusive. Using a selective high-throughput chemical screen, we discovered that the cardiac glycoside digoxin inhibits wild-type HIV-1 infection more potently than HIV-1 bearing a single point mutation (N74D) in the capsid protein. We confirmed that digoxin repressed viral gene expression by targeting the cellular Na^+^/K^+^ ATPase, but this did not explain its selectivity. Parallel RNAseq and integration mapping in infected cells demonstrated that digoxin inhibited expression of genes involved in T-cell activation and cell metabolism. Analysis of >400,000 unique integration sites showed that WT virus integrated more frequently than N74D mutant within or near genes susceptible to repression by digoxin and involved in T-cell activation and cell metabolism. Two main gene networks down-regulated by the drug were CD40L and CD38. Blocking CD40L by neutralizing antibodies selectively inhibited WT virus infection, phenocopying digoxin. Thus the selectivity of digoxin depends on a combination of integration targeting and repression of specific gene networks. The drug unmasked a functional connection between HIV-1 integration and T-cell activation. Our results suggest that HIV-1 evolved integration site selection to couple its early gene expression with the status of target CD4+ T-cells, which may affect latency and viral reactivation.

## Author summary

HIV-1 integrates more frequently within transcribed host genes, however we do not understand the biological significance of this. We found that a drug called digoxin inhibits wild type HIV-1 more potently than an HIV-1 bearing a single point mutation in the capsid protein. Here we show that digoxin represses HIV-1 gene expression and in parallel inhibits CD4+ T-cell activation and metabolism. When we analysed the integration sites of wild type and mutant HIV-1, we discovered that wild type virus integrates within or near genes involved in CD4^+^ T-cell activation and metabolism more often than the mutant virus. Because these are the very same genes repressed by digoxin, the integration bias of wild type virus makes it more susceptible than mutant virus to silencing by the drug. Digoxin unmasked a functional link between HIV-1 integration and T-cell activation, which may affect HIV-1 latency and reactivation.

## Introduction

HIV-1 infects immune cells expressing CD4 and CCR5/CXCR4, which serve as HIV-1 receptor and co-receptor for entry, such as helper T-lymphocytes and macrophages. Upon entry and reverse transcription, the virus must access the nucleus of infected cells and integrate into host chromosomes, however little is known about the steps occurring between nuclear entry and integration. The HIV-1 capsid protein (CA) was shown to be a dominant determinant of nuclear import [1]. Several studies have since indicated that CA is involved in nuclear entry and post-nuclear entry events (reviewed in [2]). CA interacts with nucleoporins Nup153 [3,4,5,6] and Nup358, via the CypA binding domain [7,8,9], although the significance of this interaction is unclear [10]. CA also interacts with the mRNA processing factor CPSF6 and with the nuclear transport receptor Transportin 3 (TNPO3), which were shown to be important for HIV-1 pre- and post-nuclear entry steps [11,12,13,14,15]. Small amounts of HIV-1 CA have been detected inside the nuclei by biochemical fractionation [15], and the antibiotic Coumermycin-A1 was shown to impair HIV-1 integration by targeting CA [16]. These observations led to the proposal that CA is involved in post-nuclear entry steps leading to efficient integration [15]. Imaging approaches have shown that nuclear CA is associated with viral nucleic acids [17,18,19] and Coumermycin-A1 was reported to block integration by preventing the completion of virus uncoating inside the nucleus [20]. HIV-1 preferentially integrates within actively transcribed genes [21,22]. Remarkably, CA binding to CPSF6 in the nucleus is critical for targeting HIV-1 integration *near* transcribed genes, whereas binding of host factor LEDGF/p75 to HIV-1 integrase seems more important for integration targeting *within* transcribed genes [23,24].

Certain CA point mutations, such as N74D, are independent of Nup358, Nup153, CPSF6 and TNPO3 for infection, and show a different integration site selection compared to wild type virus [7,23,25]. This indicates that a single point mutation in CA can change the usage of HIV-1 host factors during the early steps of infection, resulting in an altered integration pattern. However the functional significance of this altered integration distribution is unclear.

To gain greater insight into these steps of the HIV-1 life cycle, we have taken advantage of the differences between wild type (WT) and N74D mutant CA to design a novel high through put screening assay whereby CD4+ T-cells are co-infected with WT and N74D single cycle HIV-1 vectors, which are identical except for the CA point mutation. The WT virus expresses GFP whereas N74D expresses mCherry. Compound libraries are screened to find selective hits that inhibit WT more than N74D. These hits are then used to identify target molecules that affect—directly or indirectly—the interaction between host factors and HIV-1 CA and impair early events of infection.

Here, using this novel screening approach, we found that the cardiac glycoside digoxin inhibits WT more potently than N74D virus. We confirmed that digoxin inhibited viral gene expression by targeting the Na^+^/K^+^ ATPase [26] but this did not explain its selectivity. To determine the basis for the selectivity of digoxin we performed, in parallel, RNAseq and high-throughput integration site analysis in cells treated with the drug. The results showed that digoxin down-regulates genes involved in T-cell activation and cell metabolism. We found that WT virus, but not N74D virus, preferentially integrates into such genes and is affected by their repression. Therefore, using digoxin to acutely perturb specific gene expression pathways, we uncovered a functional link between HIV-1 integration preference, viral gene expression and T-cell activation and metabolism.

## Results

### Digoxin inhibits infection by WT virus more potently than N74D virus

**Fig 1:**
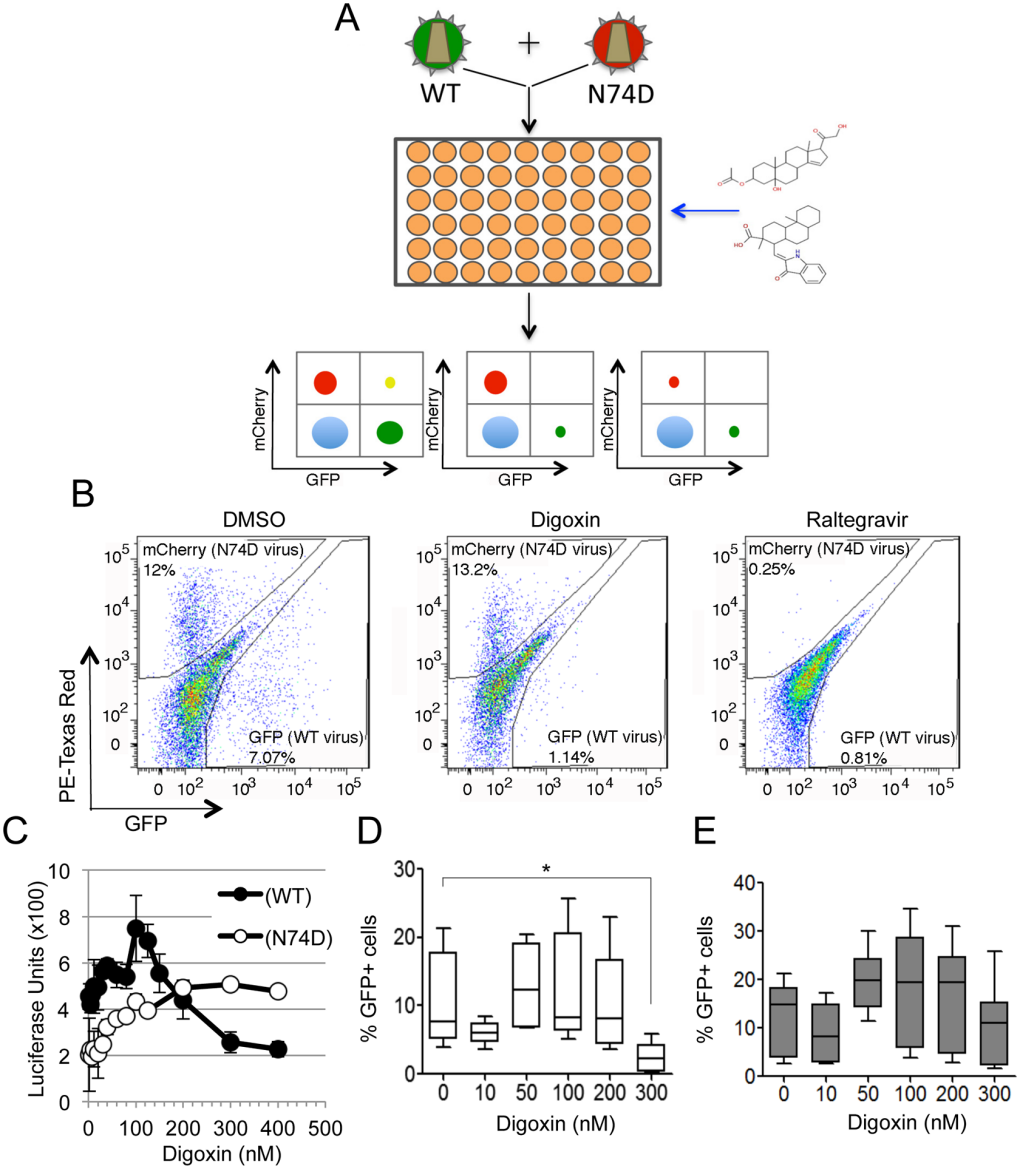
Digoxin inhibits HIV-1 infection more potently than N74D virus. (A) Schematic depiction of the high through put screen; WT HIV-1 vector expresses GFP and N74D mutant HIV-1 vector expresses mCherry. Jurkat cells are co-infected with both viruses and a library of small compounds is screened in 384 well plates. Cells are analysed by dual-colour flow cytometry. Left plot depicts a non-hit compound, middle plot depicts a selective hit and the right plot depicts a non-selective hit. (B) Jurkat cells were co-infected with WT and N74D vectors at an MOI of 0.1 in the presence of the indicated compounds and analysed 24h later. Representative dot plots from the FACS analysis for control (DMSO only), digoxin (300nM) or raltegravir (100nM) treated cells. (C) 1G5 Jurkat luciferase indicator cells were infected at the same MOI with NL4.3 WT or with NL4.3 N74D in the presence of the indicated concentrations of digoxin. Luminescence was measured 48 hours after infection; data are representative of two experiments performed in triplicate. Error bars represent SEM. (D, E) Memory CD4^+^ T-cells were stimulated via CD3/CD28 Abs for 3 days and infected with VSV-G pseudotyped HIV-1 LAIΔenv WT (D) or N74D mutant (E), cells were cultured in media containing IL-2 in the presence or absence of the indicated doses of digoxin for additional 40 hours. The percentage of viable, infected (GFP+) cells was determined by flow cytometry. Bar graphs represent mean ± SD of 9 donors. Friedman test p-values are indicated on the graphs: *, p<0.05.

We designed a novel high through put screening workflow to investigate the early events of HIV-1 replication that are influenced by CA (Fig 1A). Jurkat CD4^+^ T-cells were co-infected with two VSV-G pseudotyped single cycle HIV-1 vectors, one containing WT CA and expressing GFP (WT-GFP), and another containing the CA N74D mutation and expressing mCherry (N74D-CHE). We aimed to identify small compounds that inhibited WT virus infection at least 50% of control (DMSO) with little inhibition of N74D virus infection ("selective hits"). The rationale for this assay was based on the different usage of host cell factors by WT and N74D viruses [4,7,27]: selective hits were likely to target either the WT CA protein itself or host cell factors interacting preferentially with WT over N74D virus, directly or indirectly. After optimization, the assay had a Z' score of ≅0.7, and the number of cells infected with each type of virus, and cell survival, were both determined 24 hours post-infection by dual colour flow cytometry. The National Institute of Neurological Diseases and Stroke small-compound library was used to perform the screening at a final concentration of 1 μM (S1 File). This library contains 1041 compounds and includes many FDA-approved drugs. We detected eighteen selective hits belonging to different chemical and pharmacological classes (S1 File and S1 Table): four were anti-inflammatory, hinting that WT and N74D virus may be differentially susceptible to innate immune responses [28] and two were cardiac glycosides sharing a similar chemical structure: digoxin and digitoxin.

We focused on the cardiac glycosides because of the low probability of identifying by chance two hits with the same phenotype that share a similar chemical structure. Furthermore, digoxin was reported previously to exert potent antiretroviral activity by reducing HIV-1 gene expression [29,30], validating our screening. However, because of the different design, previous screenings did not detect the CA-dependent selectivity of digoxin, which is the focus of this study.

To confirm our screening results, we re-examined the selectivity of digoxin in single cycle infectious assays using WT-GFP or N74D-CHE HIV-1 vectors. Digoxin inhibited WT more than N74D virus whereas the integrase inhibitor raltegravir inhibited each virus equally (Fig 1B). To control that the observed phenotype did not depend on the specific fluorescent marker expressed by each vector (GFP or CHE), we reversed the markers and infected Jurkat cells with WT-CHE and N74D-GFP vectors. First, we titrated both vectors in the absence of digoxin to determine the linear range of infection and chose an MOI of 0.1–0.2 (S1A Fig). Then we infected Jurkat cells in the presence of digoxin (S1B Fig). In agreement with our previous results, the N74D vector was less sensitive to digoxin relative to WT (S1B Fig). Hence the selective response to digoxin was independent of the fluorescent marker. We sought to confirm digoxin selectivity using a replication competent virus. To this end, a titration was performed on the luciferase Jurkat reporter cell line (1G5 cells) [31] using HIV-1 NL4.3 WT or N74D, which showed that WT NL4.3 replicated marginally better than NL4.3 N74D (S1C Fig). Next, we tested the effect of digoxin by titrating the drug on 1G5 cells infected with either NL4.3 WT or NL4.3 N74D and reading luciferase 48 hours post-infection, when cell toxicity and differences in replication between the two viruses were both minimal. In these conditions, WT NL4.3 was less sensitive to digoxin than NL4.3 N74D (Fig 1C). We also tested the selectivity of digoxin in primary memory CD4+ T-cells. PBMCs were obtained from 9 healthy donors, memory T-cells (CD3+ CD4+ CD45RA-) were sorted by negative selection, stimulated using CD3/CD28 Abs and infected at an MOI of 0.1 with a VSV-G pseudotyped single cycle HIV-1 LAI with a deletion in *env* and expressing GFP in place of nef (HIV-1 LAIΔenv) [1]. Thirty-six hours post-infection, the percentage of GFP+ cells was measured by flow cytometry after gating for the live cell population. In the absence of digoxin, WT and N74D HIV-1 LAIΔenv viruses showed similar infection levels (S1D Fig). In the presence of digoxin, WT HIV-1 was inhibited more strongly than N74D at concentrations greater than 200 nM (Fig 1D and 1E), in agreement with the results in Jurkat cells. Therefore, we concluded that the high-throughput screen identified digoxin as a genuinely selective antiretroviral.

### Identification of the digoxin target responsible for antiretroviral activity in CD4+ T-cells

**Fig 2:**
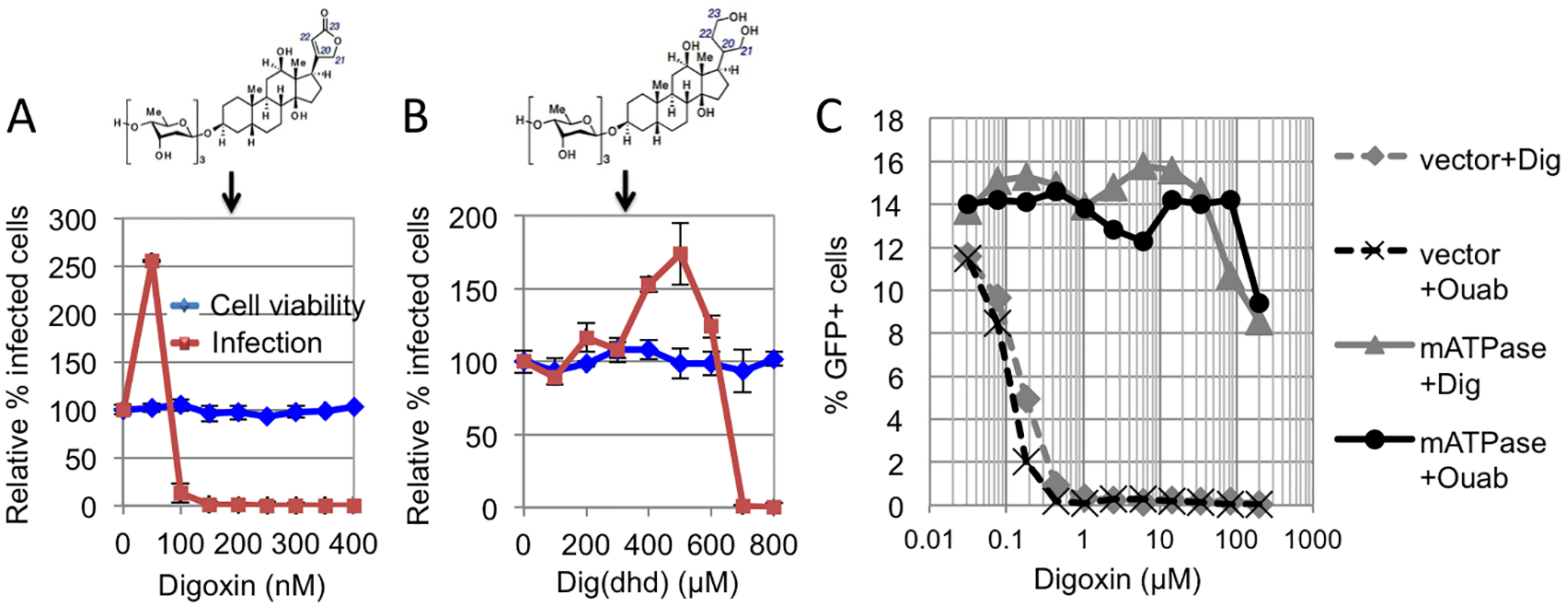
Na^+^/K^+^ ATPase is the digoxin target mediating antiretroviral activity in CD4+ T-cells. (A) Jurkat cells were infected with VSV-G pseudotyped HIV-1 LAIΔenv_GFP_ at an MOI of 0.1 in the presence of the indicated doses of digoxin and analysed by flow cytometry 36 hours post-infection. The chemical structure of digoxin is shown at the top. (B) Same as for panel (A) but cells were infected in the presence of Dig(dhd). Dig(dhd) chemical structure is shown at the top. In panels A and B error bars indicate SD, N = 3 independent experiments. (C) Jurkat cells stably expressing the mouse Na^+^/K^+^ ATPase (mATPase), or an empty vector (vector), were infected with HIV-1 LAIΔenv_GFP_ at an MOI of 0.1 in the presence of the indicated concentrations of cardiac glycosides digoxin or ouabain, both Na+/K+ ATPase antagonists. The percentage of infected, GFP+ cells was determined by FACS 24h post-infection. Data are representative of 3 independent experiments.

A previous report showed that the Na^+^/K^+^ ATPase is the main target of digoxin mediating the antiretroviral effect in 293T cells [30]. However, digoxin has two known targets, the nuclear hormone receptor RORγ/γt, (also known as RORC), which is expressed in a subset of CD4+ T-cells and innate lymphoid cells [32,33] and the α1-subunit of the Na^+^/K^+^ ATPase, which is ubiquitously expressed [34,35,36]. Therefore, in CD4+ T-cells, digoxin might have more than one target responsible for its antiretroviral activity. The co-crystal structure of RORγ/γt bound to digoxin showed that the sugar moiety of digoxin is critical for stabilizing the drug into the ligand-binding pocket of RORγ/γt [37]. Thus digoxin derivatives lacking two or more sugar moieties, such as digoxigenin or ouabain, will only bind to the Na^+^/K^+^ ATPase but not to RORγ/γt [36,38]; conversely, the lactone ring of digoxin is important for high-affinity binding to the Na^+^/K^+^ ATPase [34] hence modification of the lactone ring in 20,22-dihydrodigoxin-21-23-diol (Dig(dhd)) substantially reduces its affinity for the Na^+^/K^+^ ATPase but not for RORγ/γt [35,36]. We synthesized Dig(dhd) and tested it in infection assays in Jurkat cells using HIV-1 LAIΔenv. At 36h post-infection, cells were analysed by flow cytometry; digoxin showed the typical biphasic inhibitory curve, and at a concentration of 100 nM suppressed WT HIV-1 infection without affecting cell viability (Fig 2A). Dig(dhd) phenocopied digoxin, showing the same typical biphasic curve, however it showed a drastic drop in antiretroviral potency (Fig 2B).

This indicated that, in CD4+ T-cells, the main target of digoxin mediating the potent antiretroviral activity was the Na^+^/K^+^ ATPase. To further test this point, we expressed in Jurkat cells the murine Na^+^/K^+^ ATPase (mATPase), which is not susceptible to digoxin inhibition [39]. Cells expressing the mATPase, or control Jurkat cells, were infected with HIV-1 LAIΔenv in the presence of digoxin or ouabain. The antiretroviral activity of digoxin was drastically lower in Jurkat cells expressing the mATPase (Fig 2C). In contrast, digoxin and ouabain potently inhibited HIV-1 LAIΔenv infection in control cells (Fig 2C). These results established that, similar to 293T-cells [30], the Na^+^/K^+^ ATPase was the main digoxin target in CD4+ T-cells.

Targeting the Na^+^/K^+^ ATPase with digoxin did not impair HIV-1 reverse transcription (S2B Fig), nuclear entry (S2C Fig) or integration (S2D Fig) but reduced viral mRNA levels (S2E–S2G Fig), in agreement with previous reports [29,30]. Laird et al. showed that digoxin inhibits expression from a HIV-1 plasmid DNA transfected into 293T cells [30], which suggests that HIV-1 integration may not be necessary. Transfection of Jurkat cells is extremely inefficient hence to test if the selective phenotype of digoxin was maintained in the absence of integration, we titrated digoxin in Jurkat cells pre-exposed to a high dose (20μM) of raltegravir (a potent integration inhibitor [40]) and infected them with WT or N74D HIV-1 LAIΔenv (S2H Fig). In these conditions, residual viral gene expression mostly comes from non-integrated viral genomes, which are lost during prolonged passage in culture [41,42]. As expected, in the presence of raltegravir, infection by both WT and N74D viruses was reduced at 48h post-infection (S2H Fig) and was reduced much further 10 days post-infection (S2I Fig). This suggested that GFP expression at 48h mostly came from non-integrated viral genomes. Notably, in the presence of raltegravir, digoxin appeared to be less potent (IC_50_ >300nM) and was no longer selective (S2H Fig). This suggested that the selective phenotype of digoxin depends on integration.

### Digoxin represses gene pathways involved in cell metabolism and T-cell activation

The identification of Na^+^/K^+^ ATPase as the digoxin target did not readily explain preferential inhibition of WT over N74D virus. Digoxin inhibits viral gene expression (S2E–S2G Fig) and [29,30]), yet the promoter/enhancer regions (LTR) of WT and N74D viruses were identical, excluding that the selectivity could be mediated by a direct effect of the drug on the LTR.

HIV-1 WT has a greater preference to integrate within or near active genes than N74D virus [7,23] and digoxin can trigger changes in cellular gene expression [43,44]. We therefore hypothesized that if WT virus was more prone to integrate into genes whose expression was perturbed by digoxin then the virus might become more susceptible to the drug. Conversely, if the N74D virus were less prone to integrate into genes perturbed by digoxin then this virus would be less susceptible to the drug. To test this hypothesis we carried out, in parallel, global gene expression and integration site analysis on cells that were infected with WT or N74D HIV-1 in the presence of digoxin or DMSO.

We conducted three independent experiments in Jurkat cells infected with WT or N74D LAIΔenv, ensuring that infection levels were within the linear range (S1E Fig), and extracted total RNA and DNA from each sample 36h post-infection (S3 Fig). The RNA was used for RNAseq and the DNA was used to perform high-throughput integration site analysis. Total RNA was prepared for sequencing following the Illumina TruSeq mRNA protocol and sequenced on an Illumina Nextseq to yield an average of 15 million reads per sample. One sample (DMSO WT experiment 1) did not pass the RNA quality control and could not be sequenced. Following alignment and removal of PCR duplicates, normalized read counts were analysed using GeneSpring. A total of 2,826 genes were found differentially regulated (2-fold or more, p < 0.05) by digoxin (S2 File). Cluster analysis demonstrated a clear and significant distinction between treated and untreated samples but no significant distinction between cells infected with either WT or N74D viruses (S4 Fig).

**Fig 3:**
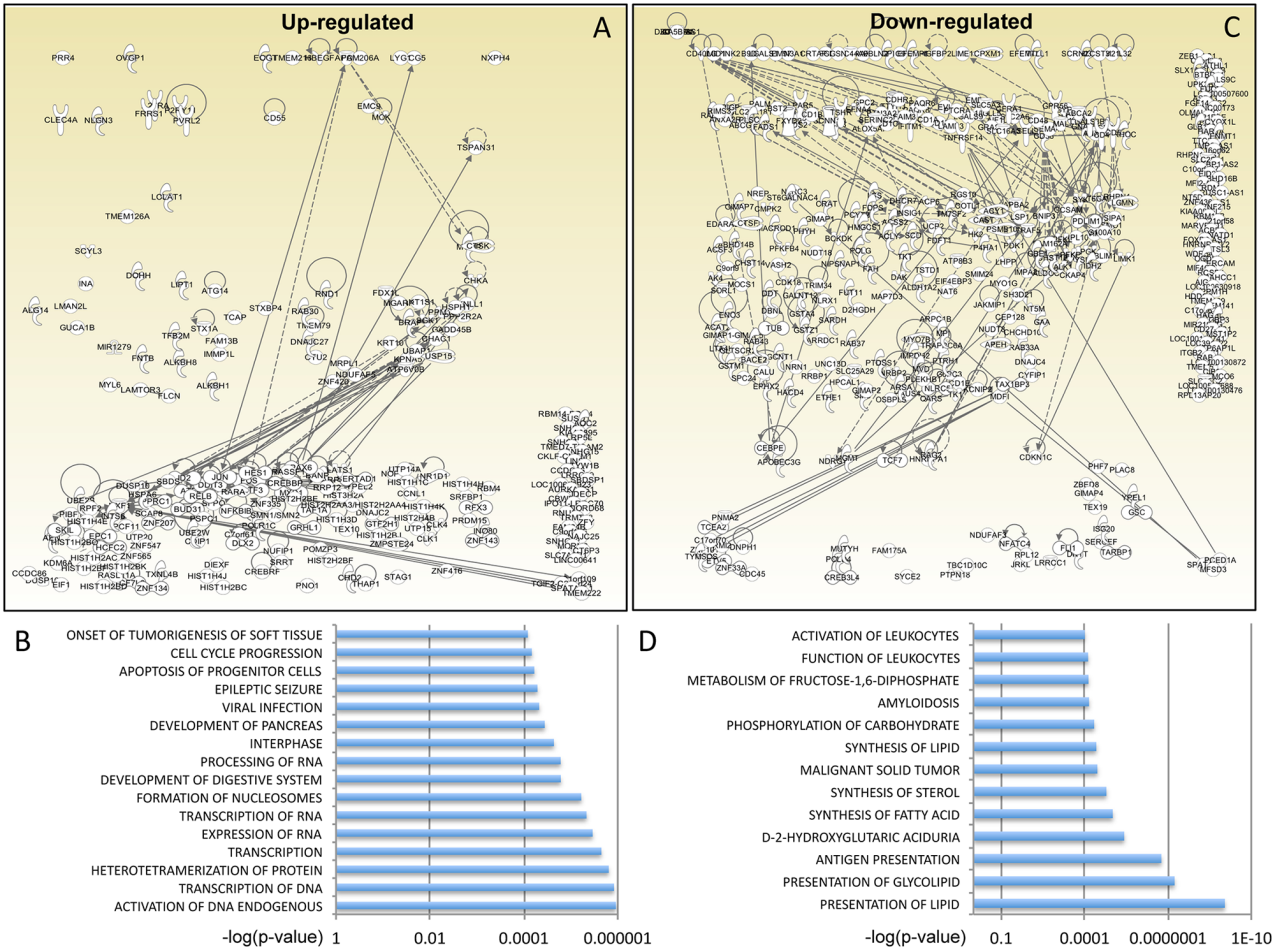
Digoxin perturbs expression of specific gene pathways. (A) IPA-generated diagram showing the 221 genes up-regulated by digoxin and their networks. Continuous lines indicate direct and experimentally validated interactions between genes; dashed lines indicate experimentally validated, indirect interactions. Circular arrows indicate self-activation. Genes that could not be assigned to a network are shown in the right hand corner. (B) List of the main functionally annotated pathways identified by IPA on the basis of the 221 genes up-regulated by digoxin. The p-value for each pathway is shown on the x-axis. The full dataset is available in S3 File. (C) IPA-generated diagram showing the 336 genes down-regulated by digoxin, same as for panel (A). (D) List of the main functionally annotated pathways identified by IPA on the basis of the 336 genes down-regulated by digoxin. The p-value for each pathway is shown on the x-axis. The full dataset is available in S4 File.

Normalised RNA read counts were studied for gene pathway enrichment using Ingenuity Pathway Analysis (IPA) (www.qiagen.com/ingenuity). To identify changes in gene expression likely to be of biological relevance, we applied a cut-off fold change ≥4 with a p-value of <0.05 (Mann Whitney test after Benjamini-Hochberg false discovery rate). Using this filter, we found digoxin up-regulated 221 and down-regulated 336 genes that could be unequivocally mapped (S2 File). Within the up-regulated gene group, IPA showed that the main biological functions affected by digoxin, in terms of both number of participating genes and significance, were transcription of RNA and DNA, chromatin remodelling, cell cycle progression and cell death and survival (Fig 3A, 3B and S3 File). A prominent up-regulated network was AP-1, including Jun, *Fos* and *ATF3*; *Jun* was one of the most up-regulated genes, increasing 60 fold in digoxin-treated cells (S2 File). Such strong up-regulation of *Jun* may be related to a survival response to stress induced by digoxin [45].

Within the down-regulated gene group, the main biological functions affected by digoxin were metabolism, in particular of cholesterol, lipids and carbohydrates, antigen presentation, T-cell signalling and activation of leukocytes (Fig 3C and 3D S4 File).

### WT HIV-1 preferentially integrates within or near genes susceptible to silencing by digoxin

To validate the hypothesis that the selectivity of digoxin occurs at the integration stage, we examined the integration profile of single cycle WT and N74D HIV-1 LAIΔenv in digoxin-treated and untreated Jurkat cells. We analysed integration site selection using a high-throughput method we recently developed [46,47]. We obtained between 15,000 and 56,000 unique proviral integration site (UIS), depending on the sample (S5A Fig and Dasaset 5). No significant differences in the number of UIS were observed between control and digoxin-treated samples (S5A Fig). This was in agreement with the Alu-LTR qPCR results, which demonstrated that digoxin did not inhibit HIV-1 integration (S2C Fig). Furthermore, no clonal expansion was detected (compare total clones to shear sites in S5A Fig) because infected cells were examined 36 hours post-infection.

**Fig 4:**
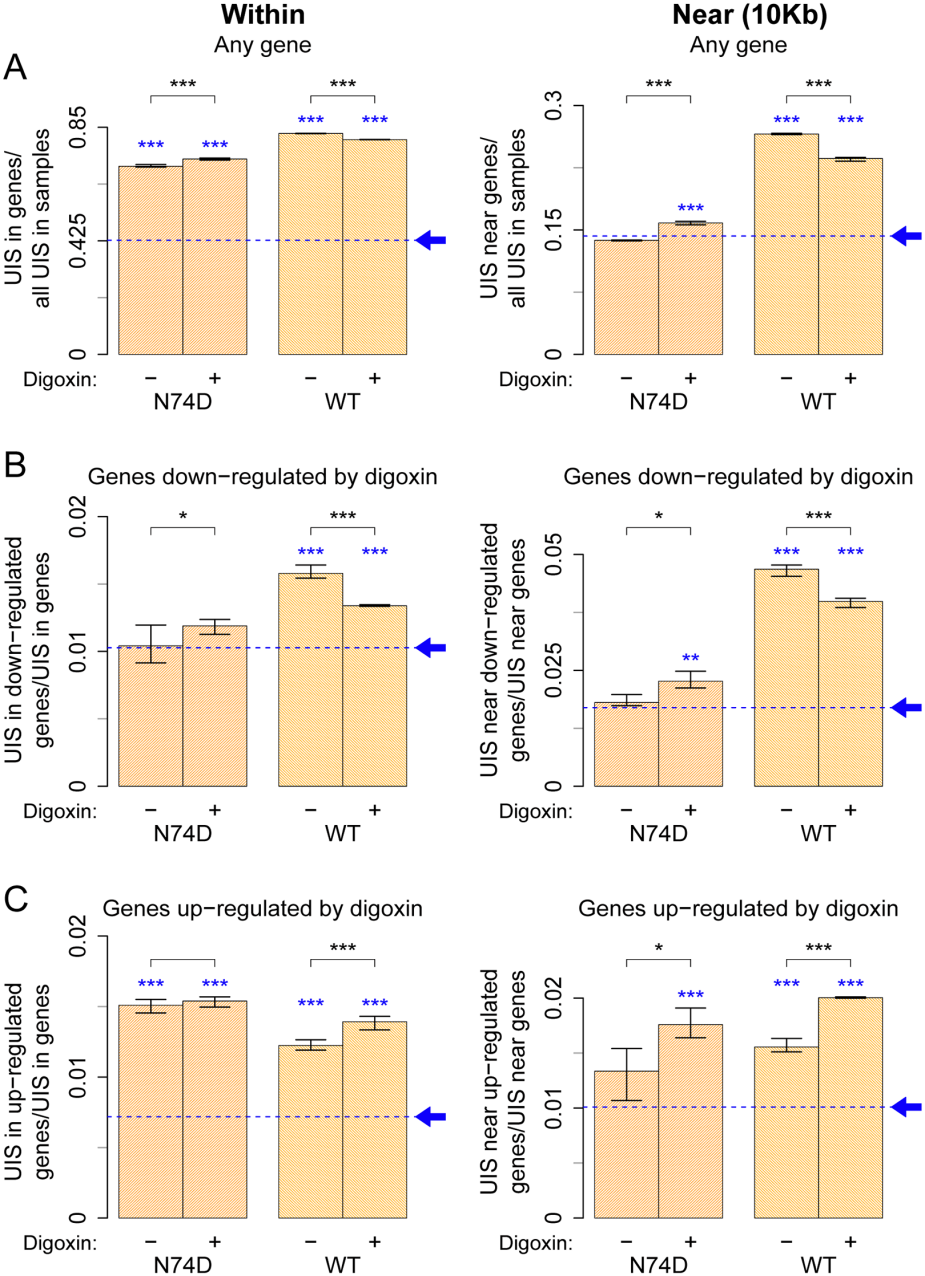
WT HIV-1 integrates more frequently within or near genes that are susceptible to down-regulation by digoxin. Three separate aliquots of Jurkat cells were independently infected with single cycle VSV-G pseudotyped HIV-1 LAIΔenv WT or N74D at an MOI of 0.2 in the presence of DMSO or 400nM digoxin. Thirty-six hours later, DNA was extracted and integration sites were identified and quantified. Same analysis was carried out for a dataset of random integration sites generated in silico, sampled from a gap-excluded reference of the human genome (hg19). This matched random control is shown as a blue dashed baseline, pointed by an arrow. (A) Plots for integration of HIV-1 WT and N74D within (left panel) or near (within 10kb of start site) (right panel) known genes. (B) Plots for integration of WT and N74D virus within (left panel) or near (right panel) genes susceptible to down-regulation by digoxin relative to their baseline bias to integrate within or near any gene. (C) Same as (B) but plots show integration within (left panel) or near (right panel) genes susceptible to up-regulation by digoxin. Each bar shows an aggregate result from all three replicates, with the range of results for the individual replicates shown by the whiskers. Black asterisks above bar graphs denote statistical significance between control (DMSO) and digoxin-treated samples. Blue asterisks just above the bar graphs denote statistical significance in the samples relative to baseline (same analysis carried on the in silico sites) and was assessed using the Fisher’s exact test with Bonferroni’s correction for multiple comparisons (* < 0.05, ** < 0.01, *** < 0.001).

UIS were mapped to human genome reference hg19 (S5 File); a control was generated in silico using 100,000 non-gap genomic positions chosen at random, which were processed in the same way as the experimentally observed UIS. WT and N74D viruses integrated preferentially within any gene, however WT virus favoured integration within any gene more strongly than the N74D virus relative to the expected random distribution (baseline) (Fig 4A left panel and S5B Fig). WT virus had a clear preference (≈2 fold above baseline) to integrate near any gene (10 Kb from the transcriptional start site) whereas N74D virus did not (Fig 4A right panel), in agreement with previous observations [7,23,25]. Digoxin reduced WT virus integration near any gene and increased N74D virus integration near any gene (p < 0.001, Fisher’s exact test) but this effect was quite small (Fig 4A). In these conditions, the orientation of the provirus relative to the cellular gene transcriptional start site was unchanged (S5C Fig). Thus digoxin caused modest, albeit selective, changes in the integration preference of HIV-1 WT and N74D virus. Nonetheless, irrespective of digoxin treatment, a significant gap remained between WT and N74D virus integration preference, which warranted further investigation.

To achieve greater specificity, we examined integration preference within or near genes whose expression was changed (≥4 fold up- or down-regulated) by digoxin, according to our RNAseq results (S2 File). For greater stringency, because of the observed bias of WT virus to integrate near and within genes, an in silico control for random integration sites within or near genes was generated, which was processed in the same way as the experimentally observed data. Then the ratio between UIS within or near genes down regulated by digoxin and all UIS within or near genes was calculated. Notably, WT virus integrated more often than N74D virus within genes susceptible to down-regulation by digoxin (≈1.5 fold above baseline) (Fig 4B left panel), and this effect was stronger (≈2.5 fold above baseline) near such genes (Fig 4B right panel), demonstrating selectivity. This difference between WT and N74D virus was large enough to suggest biological relevance. Digoxin treatment had a small yet selective impact on the integration preference of each virus (Fig 4B).

WT and N74D viruses also integrated more often than matched random control within or near genes susceptible to up-regulation by digoxin (Fig 4C). Notably, however, in contrast to the situation in the down-regulated genes, there was no WT/N74D selectivity for integration within up-regulated genes, there was a modest selectivity for integration near up-regulated genes and digoxin treatment enhanced the integration preference of each virus (Fig 4C). Thus integration within or near genes susceptible to up-regulation by digoxin was unlikely to explain the selective phenotype.

**Fig 5:**
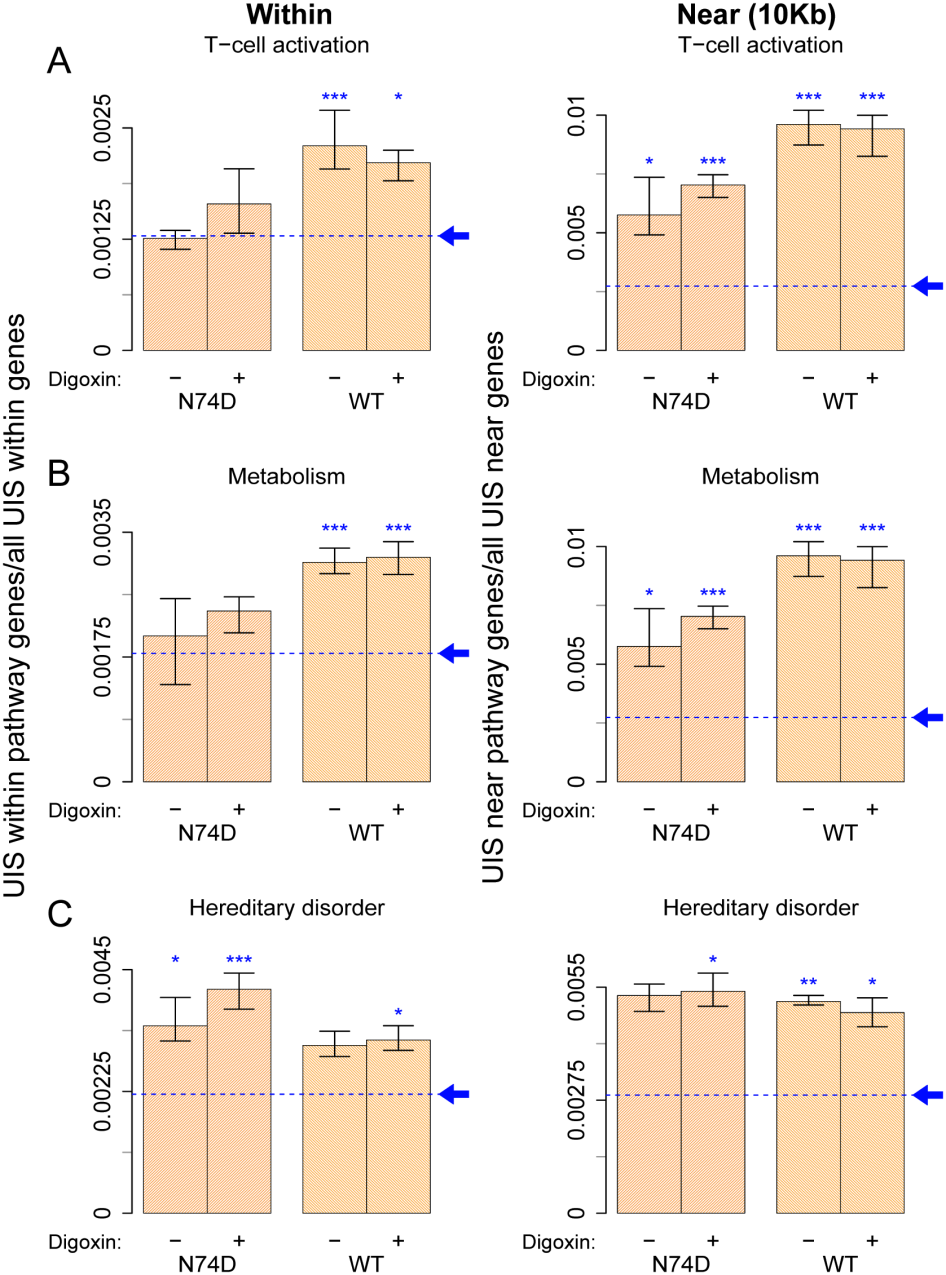
WT HIV-1 integrates more frequently than N74D within or near genes involved in T-cell activation and cell metabolism. UIS, as well as matched random controls, were obtained as described in Fig 4 and annotated for integration within or near (within 10kb of transcript start site of) known genes, as well as genes susceptible to down-regulation by digoxin belonging to specific IPA pathways: T-cell activation, cell metabolism and hereditary disorder. Plots for integration of WT and N74D virus within (left panel) or near (right panel) T-cell activation genes (A), cell metabolism genes (B) and hereditary disorder genes (C). Each bar denotes the proportion of integration sites found within (left panel) or near (right panel) genes of the particular pathway within all integration sites within or near genes. The same analysis was carried out in the matched random control sites, shown as the blue dashed baseline, pointed by an arrow. Each bar shows an aggregate result from all three replicates, with the range of results for the individual replicates shown by the whiskers. Statistical significance refers to UIS in sample relative to the baseline bias and was assessed using the Fisher’s exact test with Bonferroni’s correction for multiple comparisons (* < 0.05, ** < 0.01, *** < 0.001). No significant difference was found between control (DMSO) and digoxin-treated samples in these groups.

In light of these results, we next examined integration within or near genes belonging to the two main gene pathways down-regulated by digoxin: T-cell activation and cell metabolism, which are intimately related both to each other and to HIV-1 infection [48,49,50,51]. Based on IPA, we identified 59 and 73 genes involved in T-cell activation and cell metabolism, respectively (S4 File). In the absence of digoxin, integration of WT virus within T-cell activation genes was ≈1.8-fold above baseline, and approximately 4-fold above baseline near such genes (Fig 5A). The N74D virus integration preference within this gene group was similar to baseline whereas integration near these genes was ≈2-fold above baseline (Fig 5A). Digoxin increased N74D virus integration frequency within or near the T-cell activation genes although this did not reach statistical significance, presumably due to the lower number of integration sites that could be analysed (Fig 5A). Digoxin had little or no effect on WT virus integration frequency within or near T-cell activation genes, which remained much higher than random, irrespective of the drug (Fig 5A). To explain this observation, we speculate that WT virus integration within or near this gene group is faster than the effect of digoxin on their expression levels. A similar trend in WT and N74D virus integration preference was found for the group of genes related to cell metabolism (Fig 5B). Therefore, although a total of only 132 genes were analysed in the T-cell activation and cell metabolism pathways, WT virus integration within or near these genes was disproportionally frequent both in absolute terms and relative to the N74D virus.

As a control for this analysis, we also examined integration preference in the hereditary and developmental disorder gene network (66 genes), also identified by IPA among the genes down-regulated by digoxin (S6 File). Although there was integration preference above baseline in this group of genes, it was not as pronounced as for T-cell activation or metabolism genes and there was no difference between WT and N74D viruses (Fig 5C). This suggested that WT virus has some degree of specific integration targeting within certain groups of genes.

**Fig 6:**
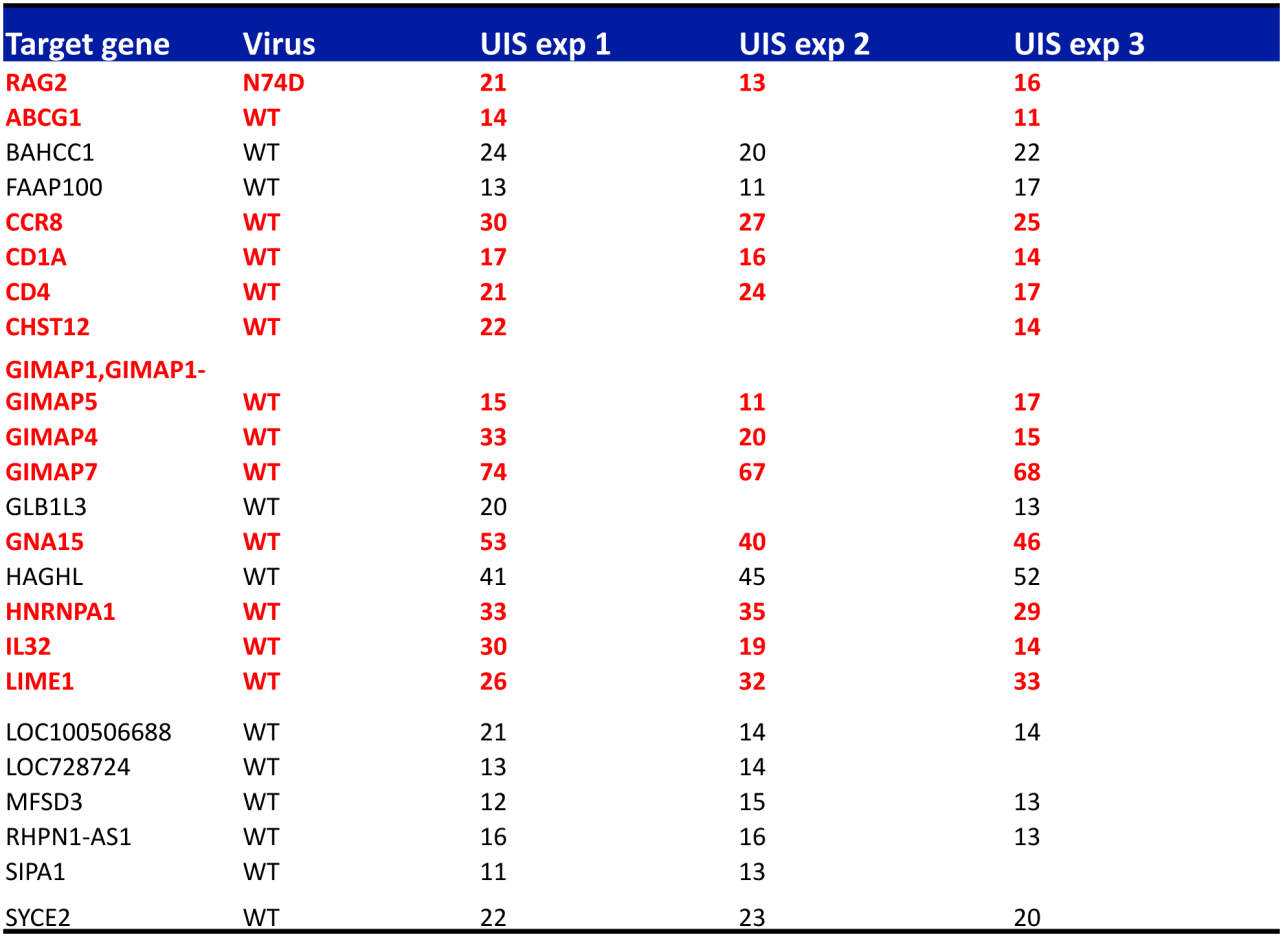
Analysis of integration frequencies shows enrichment of integration hotspots within genes involved in T-cell activation and cell metabolism. Global integration frequencies were calculated in each replicate experiment. An integration hotspot had > 5 independent integration events in at least two replicate experiments. Genes highlighted in red are involved in T-cell activation or cell metabolism as determined by IPA and Genecards annotation.

To test this notion further, we looked for integration hotspots of WT or N74D viruses in our samples. Hotspots were defined as genes having more than 5 UIS in at least two out of three experiments. We detected 24 hotspots in total with integration frequencies ranging from 11 to 74 UIS (Fig 6). Remarkably, WT virus had 23 hotspots and N74D virus had only one hotspot (in the RAG2 gene) (Fig 6). We annotated the hotspots using IPA and Genecards and found that 13 of them were in genes belonging to the cell metabolism, T-cell activation and antigen presentation pathways (highlighted in red in Fig 6). This result supported the notion that WT selectively integrates within specific gene pathways and that this selectivity is lost for the N74D virus.

**Fig 7:**
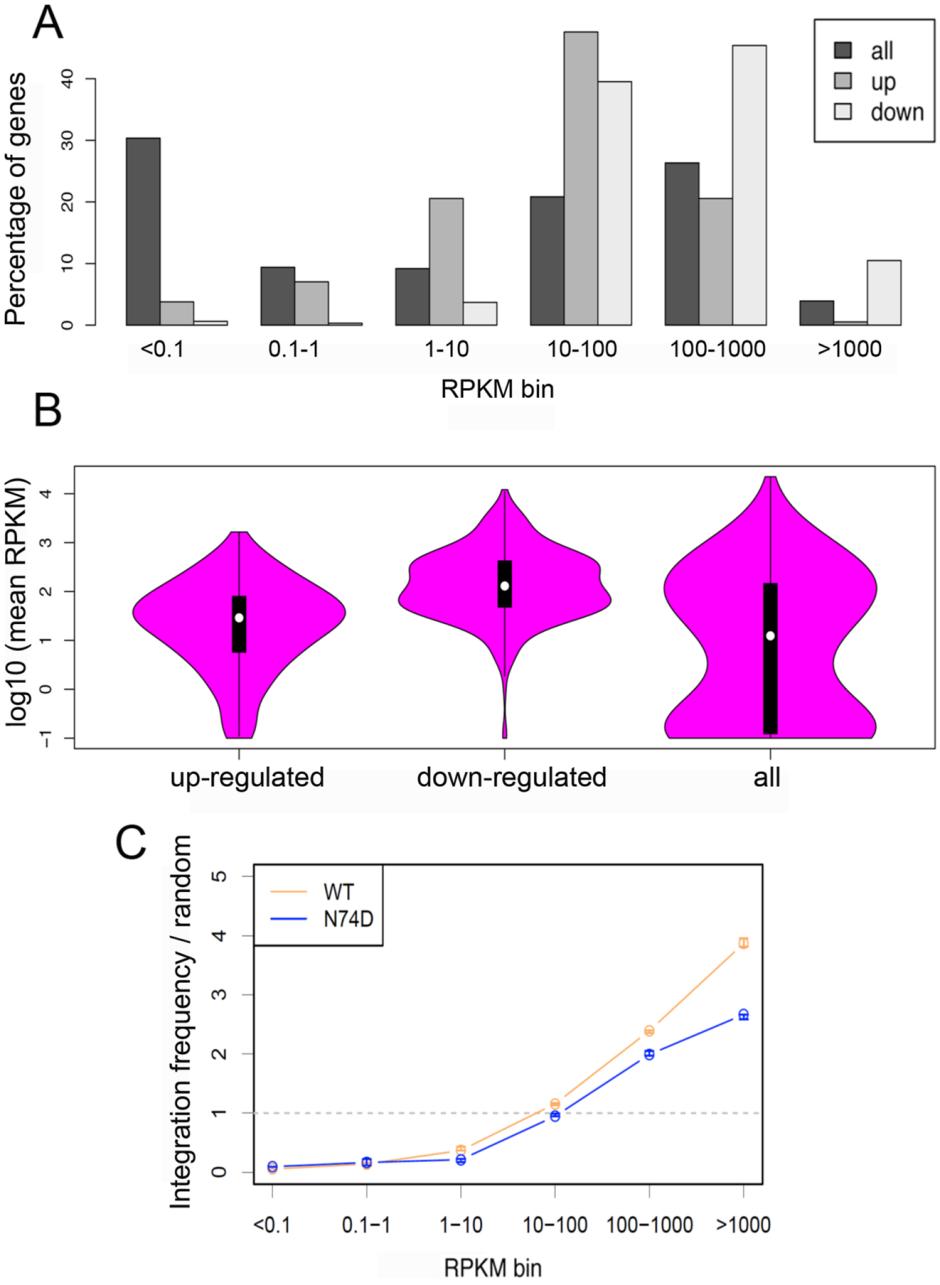
Genes sensitive to digoxin are highly expressed. (A) The percentage of genes expressed at low, intermediate or high levels were plotted for each group: all genes (n = 20100), genes up-regulated by digoxin (n = 221) and genes down-regulated by digoxin (n = 336). Normalized expression values (RPKM) for each gene in each dataset were binned according to expression intensity in the digoxin-negative samples (x-axis). For each expression bin, the percentage of genes present in that bin is shown. (B) The distribution in normalized expression (in the absence of digoxin) between upregulated, downregulated or all genes. (C) Correlation of integration frequency with measured levels of gene expression. Normalized expression values (RPKM) for each gene in each dataset were binned according to expression intensity (x-axis). Y-axis indicates UIS in a gene of a particular expression bin (normalized by gene length) over the proportion of random sites in genes in the same bin. Each circle on the graph is the median of three experiments, and the whiskers indicate the range of values observed over the three experiments.

Recent studies showed that HIV-1 tends to integrate more frequently within highly expressed genes [23,52] hence the selective integration preference of WT virus might be influenced by expression levels of target genes. We compared expression of the genes susceptible to up- or down-regulation by digoxin with that of all genes (Fig 7). Genes unaffected by digoxin showed a biphasic distribution with approximately 30% of them little or not expressed and approximately 25% of them highly expressed (Fig 7A). Genes susceptible to down-regulation by digoxin were the most highly expressed, followed by genes susceptible to up-regulation by the drug. Furthermore, the median level of expression was significantly higher for genes susceptible to down-regulation compared to the other two groups (Fig 7B). We correlated gene expression levels with frequency of integration for WT and N74D viruses. There was a clear correlation between gene expression and integration frequency for both WT and N74D viruses, however we detected selectivity in the most highly expressed gene group: WT virus integrated into such genes more frequently than N74D virus (Fig 7C), which is in agreement with a recent report [23]. Thus WT virus integrates more frequently than N74D virus within or near the most highly expressed genes; genes susceptible to down regulation by digoxin are amongst the most highly expressed hence WT virus integrates into such genes more frequently than N74D virus. These results support the link between differential sensitivity to digoxin of WT and N74D viruses and their different integration preference.

### Inhibition of CD40L selectively affects WT HIV-1 infection

**Fig 8:**
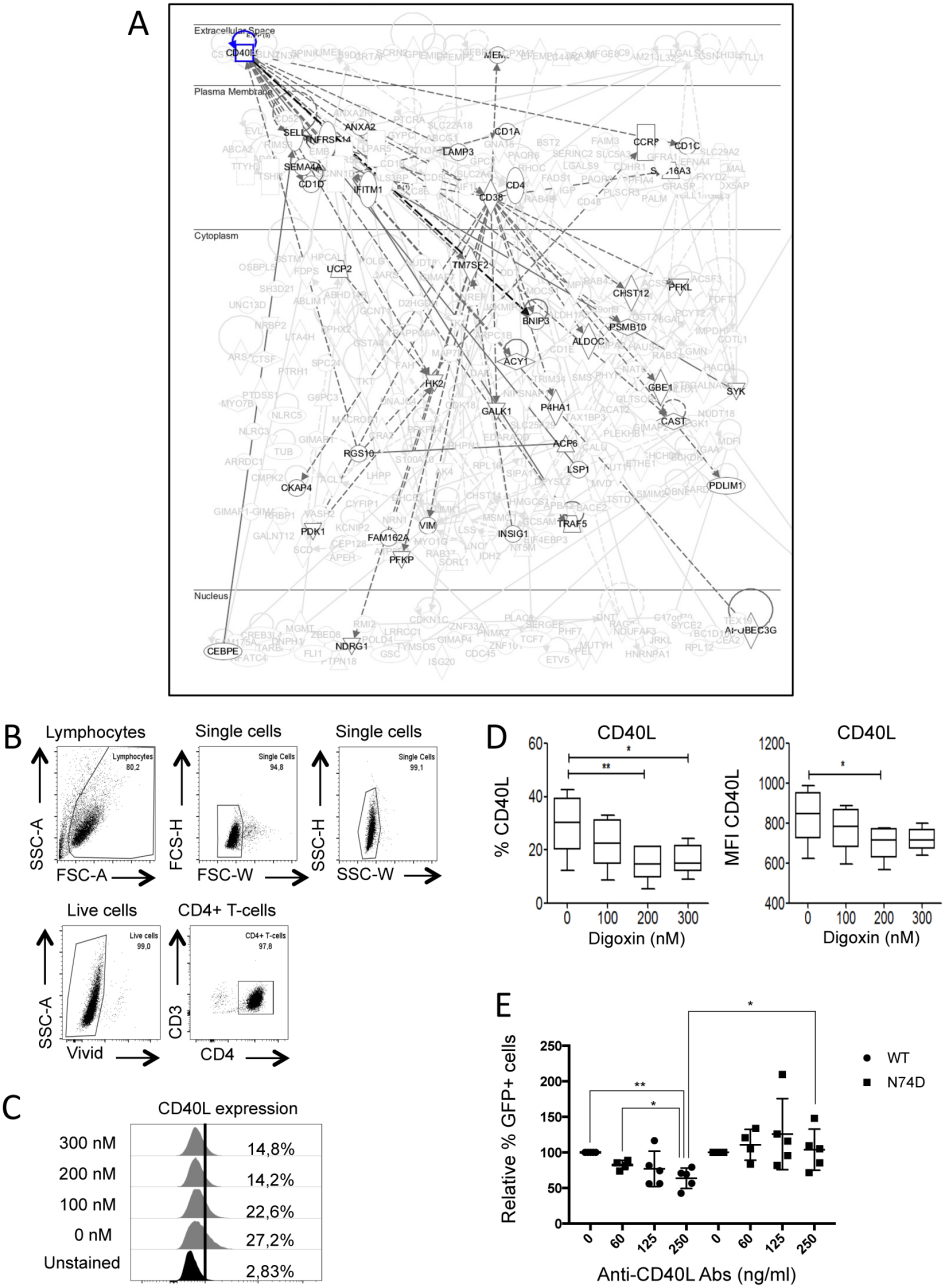
Inhibition of CD40L selectively affects WT HIV-1. (A) IPA diagram highlighting genes in the CD40L pathway that are susceptible to down-regulation by digoxin. HIV-1 WT preferentially integrates within or near these genes. Continuous lines indicate direct and experimentally validated interactions between genes; dashed lines indicate experimentally validated, indirect interactions. (B-D) Purified memory CD4+ T-cells were stimulated *via* CD3/CD28 Abs, cultured for 3 days and exposed to the indicated concentrations of digoxin for 24h. Cells were labeled with anti-CD40L FITC-conjugated antibodies, stained for cell viability and analyzed by flow cytometry. (B) Gating strategy to select single, live CD4+ T-cells. (C) Representative plot showing the percentage of memory CD4+ T-cells expressing CD40L on their surface in the presence of the indicated concentrations of digoxin (y-axis). (D) The percentage of CD40L positive cells and the MFI were quantified by flow cytometry. Bar graphs represent mean ± SD of 3 donors. Friedman test p-values are indicated on the graphs: *, p<0.05; ** p<0.01. (E) Jurkat cells were infected with HIV-1 LAIΔenv _GFP_ WT or N74D at MOIs of 0.05–0.2 in the presence of the indicated concentrations of the anti-CD40L neutralizing antibody and analysed by flow cytometry 48h after infection to measure the percentage of GFP+ cells. Dot plots showing the results of five independent experiments. Unpaired, two-tailed t-test p-values are indicated on the graphs: *, p<0.05; **, p<0.01.

We sought to test further the biological relevance of this link. Within the T-cell activation pathway identified by IPA, the CD38 and CD40L signalling networks were prominent and showed some degree of overlap (Fig 8A and S6A Fig). Upon digoxin treatment, RNAseq showed that CD40L down-regulation was 8-fold and CD38 down-regulation was 4-fold (S2 File). T-cells express CD38 and CD40L upon activation and proliferation [53,54] and lower levels of these markers in digoxin-treated cells suggested that the drug might induce a more quiescent phenotype. Given the profound impact of T-cell activation on HIV-1 gene expression [48] and the high frequency of HIV-1 integration within or near genes of this network, we chose the CD40L pathway for further analysis. We isolated primary CD4+ memory T-cells from 3 healthy donors, stimulated them with anti-CD3/CD28 beads, treated them with digoxin for 24h and analysed cells by flow cytometry for surface expression of CD40L. Digoxin consistently reduced levels of CD40L, both in terms of number of cells expressing the marker and their mean fluorescent intensity (MFI) (Fig 8C and 8D), validating the RNAseq data. Digoxin also down-regulated CD38 expression in primary CD4+ T-cells (S6B and S6C Fig)

We then blocked CD40L with a neutralizing antibody in acutely infected cells. Jurkat cells, which express functional CD40L [55,56], were infected with HIV-1 LAIΔenv WT or N74D at an MOI ranging from 0.05 to 0.2 in the presence of different doses of an anti-CD40L neutralizing antibody. Cells were analysed 36-48h post-infection; the neutralizing antibody reduced WT HIV-1 infection in a dose-dependent way, with maximal inhibition at 250ng/ml (Fig 8E). Remarkably, the N74D virus was unaffected by the antibody (Fig 8E). Thus inhibition of CD40L by the anti-CD40L neutralizing antibody had a selective effect on HIV-1 infection, mimicking digoxin. Overall our results support the notion that the virus integration preference, combined with targeted gene repression, had functional consequences.

## Discussion

Using a novel screening approach, we found that digoxin inhibited WT HIV-1 more potently than the N74D mutant virus. We confirmed previous studies showing that digoxin represses HIV-1 gene expression, yet WT and N74D viruses share an identical promoter. We found that digoxin repressed preferentially cellular genes involved in T-cell activation and cell metabolism. We subsequently found that WT HIV-1 integrated within or near those genes more frequently than N74D HIV-1. This led us to hypothesize that the integration preference of WT virus makes it more susceptible to digoxin than N74D virus because WT virus would be repressed in tandem with cellular gene expression. To our knowledge, our results for the first time demonstrate a functional connection between acute changes in T-cell activation or metabolism and HIV-1 integration site selection. We suggest that this connection has important biological significance.

It was previously reported that digoxin has potent antiretroviral activity [29,30] and that the drug specifically impairs the generation of single-spliced viral mRNAs and production of Rev [29]. In our screen, we used HIV-1 vectors that express GFP from an unspliced mRNA yet an inhibitory effect of digoxin was still detected. Furthermore, our WT and N74D HIV-1 constructs were identical except for the point mutation in CA. Thus it is unlikely that splicing defects of the viral mRNA are the main cause of the digoxin phenotype observed in the present study. Accordingly, Wong et al. also observed that HIV-1 mRNA splicing defects, or Rev inhibition, were insufficient to fully explain the potent antiretroviral activity of digoxin [29]. We note, however, that the IC_50_ of digoxin reported by Wong et al. [29] is lower than the IC_50_ reported in the present study. This indicates that our single round infection assays might bypass the viral gene expression block caused by aberrant splicing. Indeed, in our LAIΔenv HIV-1 constructs, Nef is replaced by GFP, which is expressed from a multiply spliced viral RNA independent of Rev for export [1]. Laird et al. [30] found that digoxin can also inhibit expression from an HIV-1 plasmid transfected in 293T cells, suggesting that integration may not be necessary. Because transfection of Jurkat cells is extremely inefficient, we infected cells in the presence of digoxin and a high dose of raltergavir to inhibit integration. In these conditions, GFP expression mostly comes from non-integrated viral genomes, which are lost during prolonged passage in culture [41,42]. Raltegravir produced two effects: it increased digoxin IC_50_ above 300nM and abrogated digoxin selectivity. These results suggested that integrated proviruses are more susceptible to digoxin than non-integrated viral genomes and that the selectivity of digoxin depends on integration. This is unsurprising because integrated proviruses are chromatinized and become susceptible to more complex regulatory mechanisms than non-integrated HIV-1 genomes [48,57,58].

In any case, our present study focuses on the selective activity of digoxin, which is novel and was discovered because of the unique design of our screen. By concentrating on the mechanism that underpins digoxin selectivity, we have unmasked a link between CD4 T-cell activation and HIV-1 integration of broad significance.

Because WT and N74D viruses were identical, except for the point mutation in CA, the selective activity of digoxin was unlikely to have a direct explanation. Instead, our results suggest that selectivity may result from a combination of factors. Firstly, in normal conditions, WT and N74D viruses have different integration site distributions and we found that WT virus integrates more frequently than N74D virus within or near genes implicated in T-cell activation and cell metabolism, which are highly expressed in activated CD4+ T-cells. Secondly, as it happens, we found that digoxin inhibits specific gene pathways, mostly involved in cell metabolism and T-cell activation. The simplest interpretation linking these results together is that WT virus, by virtue of its integration preference, is intrinsically more susceptible than N74D virus to transcriptional perturbations of certain genes (S7 Fig).

This interpretation is supported by the differential effect of blocking CD40L on WT and N74D viruses, which mimicked digoxin. The selective effect of CD40L blockage would be difficult to explain with a direct effect on the viral promoter. Instead, the results suggest that a proportion of the GFP signal in cells infected with WT virus is accounted for by proviruses susceptible to CD40L blockage. It is unclear if proviruses integrated within or near genes belonging to specific pathways such as cell metabolism and T-cell activation are more highly expressed, or more likely to be expressed in T-cells. Our results hint at the possibility that certain networks of genes, in which HIV-1 preferentially integrates, may contribute disproportionally to viral gene expression at the early stages post-infection, although further work will be necessary to address this issue. It would also be interesting to investigate if the selective digoxin effect on HIV-1 gene expression is recapitulated in other cell types. We speculate that the selective phenotype might be smaller because genes pathways for T-cell signalling would not be highly expressed, although presumably gene pathways for metabolic activity would be, depending on the cell type.

Our interpretation to explain the selectivity of digoxin requires that HIV-1 gene expression should be influenced by the status of the surrounding chromatin, whereby locally induced chromatin repression also affects the provirus. In support of this notion, it has been shown that the genomic site of provirus integration influences HIV-1 transcription before sufficient amounts of viral protein Tat are made (the so-called Tat-independent phase) [59]. Furthermore, chromatin repression near or at the newly integrated provirus prevents the remodelling at the LTR DNA necessary for viral transcription [60,61,62], until Tat promotes chromatin activation [63].

It is presently unclear how CA should influence the activity of a compound acting on viral gene expression. Nonetheless, because the selective phenotype of digoxin depends at least in part on integration site selection, it should be influenced by specific interactions between CA and host factors that target HIV-1 integration. One candidate is CPSF6, which was recently reported to direct HIV-1 integration near highly expressed, intron-rich and highly spliced genes [23]. Initial experiments using 293T CPSF6 KO cells [23] did not show a major role for CPSF6 in mediating the selective phenotype of digoxin, clearly though CD4+ T-cells would be more relevant targets. We have been unable so far to generate CPSF6 knock down or KO CD4+ T-cells. It is also possible that additional host factors, such as LEDGF/p75, cooperate with CPSF6 to direct HIV-1 integration into genes implicated in T-cell activation and metabolism. The degree of proximity of WT and N74D integration sites to heterochromatin located at the nuclear periphery may also be important to determine susceptibility to digoxin [64,65]. These lines of investigation are worth pursuing in the future.

It is well established that HIV-1 preferentially integrates within transcribed, intron-rich and highly spliced genes embedded with histone marks of active chromatin [21,22,23,24,52]. However, the functional significance of this targeting has remained rather elusive. By acutely perturbing cellular gene expression and comparing WT to N74D mutant viruses, we have been able to show that, in CD4+ T-cells, WT HIV-1 has a significant greater preference to integrate into or near genes implicated in T-cell activation and cell metabolism than N74D virus, which has functional consequences at least in the early stages post-infection. Our analysis is limited to a CD4+ T-cell line. Nonetheless, the overall pattern of HIV-1 integration site selection is conserved across many different cell lines and primary cells [66,67,68,69,70] and we detected the selective effect of digoxin on HIV-1 infection in primary memory CD4+ T-cells too. It is therefore likely that our observation is true in many cell types, including primary cells.

We suggest that a functional link connecting HIV-1 integration preference to T-cell activation and metabolism has broad significance. Activation of naive T-cells, their differentiation into quiescent memory cells and the activation of memory cells are rapid events that require profound physiological changes involving specific T-cell signalling pathways and metabolic adaptation [49,50,71]. The metabolism of CD4+ T-cells changes dramatically on entering or exiting quiescence: upon activation, T-cells adopt an anabolic metabolism, increase glycolysis and fatty acid oxidation whereas quiescent cells rely mainly on oxidative phosphorylation [71]. Because the site of provirus integration influences early HIV-1 transcription [59], our results suggest that the probability of HIV-1 integration within or near genes involved in T-cell activation and cell metabolism may favour rapid viral gene expression and replication in activated CD4+ T-cells. Conversely, if a CD4+ T-cell enters quiescence shortly after infection, proviruses within or near T-cell activation and metabolic genes should be more likely to become silenced in tandem with the cellular genes due to changes in the local chromatin. Therefore the cell fate (quiescent/memory or activated/effector) would be linked to the virus fate (silenced or active) for a short time post-infection, before Tat overrides local chromatin constraints to HIV-1 gene expression [72]. In this scenario, in cooperation with transcription factors expressed in CD4+ T-cells that activate the viral promoter [48], integration site selection would be an additional layer regulating HIV-1 gene expression in a cell-state dependent manner. Consistent with this idea, it was recently shown that retargeting HIV-1 integration in CD4+ T-cells away from actively transcribed genes by LEDGF/p75 antagonists reduces the fraction HIV-1 that can later be reactivated from latency [73].

## Materials and methods

### Ethics statement

Blood samples were obtained from healthy volunteers after written informed consent according to the approved protocol of the UCL Ethics Committee ref. 2649/001. The study was approved by the joint University College London / University College Hospitals National Health Service Trust Human Research Ethics Committee and written informed consent was obtained. For experiments shown in Fig 1 and Fig 8, peripheral blood mononuclear cells (PBMCs) were collected at the McGill University Health Centre, Montreal, Quebec, Canada from healthy donors, in compliance with the principles included in the Declaration of Helsinki and received approval from the Institutional Review Board of the McGill University Health Centre and CHUM-Research Centre. All subjects signed a written informed consent for their study participation.

### Chemical compounds

Digoxin, ouabain, coumermycin-A1, and nevirapine were obtained from Sigma-Aldrich, Dorset, UK; raltegravir was obtained from Santa Cruz Biotechnology, Dallas, TX, USA, digitoxin was obtained from MP Biomedicals, UK, digoxigenin from Fluka, Dorset, UK. 20,22-dihydrodigoxin-21-23-diol (DHD) was synthesized as previously described [36].

### Cell lines and viruses

HeLa and 293T cells (European Collection of Authenticated Cell Culture [ECACC] Public Health England, UK) were grown in Dulbecco’s modified Eagle’s medium (DMEM) (Gibco Labs, Paisley, UK) supplemented with 10% foetal calf serum (FCS) (Helena Bioscience, Newcastle, UK) and 2 mM glutamine (Gibco Labs) at 37°C in 5% CO_2_. Jurkat E6.1 (ECACC) and Jurkat indicator line 1G5 containing the firefly luciferase gene driven by the HIV LTR (AIDS Research and Reference Reagent Program, Division of AIDS, NIAID, NIH: from Dr. Estuardo Aguilar-Cordova and Dr. John Belmont) were grown in RPMI medium (Gibco Labs) supplemented with 10% FCS at 37°C in 10% CO_2_. Viral stocks were prepared by transfection of 293T cells as described previously [15,74] using pHIV LAIΔenv (gift of Michael Emerman, Fred Hutchinson Cancer Research Centre, Seattle, USA) [1], HIV-1_GFP_ vectors (gift of Adrian Trasher, Institute of Child Health, UCL, UK) and NLENG1-IRES YU-2 (HIV-1 modified to contain GFP and Nef separated by an IRES–[75] (gift of David N. Levy, New York University, USA). HIV isolates NL4.3 (Centre for AIDS Reagents, Health protection Agency, UK), YU-2 and BaL (NIH AIDS Reagent Program) were produced by Fugene transfection into 293T cells and supernatant containing viral particles was collected 48h post-transfection as described [15,16]. Reverse transcriptase (RT) activity was measured by the Lenti-RT^™^ Activity Assay (Cavidi Tech, Uppsala, Sweden) following the manufacturer’s instructions. For infections, 13ml of 1G5 cells (~0.9x10^6^/ml) were mixed with 2ml NL4-3 (WT or N74D). The mix was dispensed robotically, 45μl/well onto 384-plates preloaded with drug dilutions. Samples were analysed 48 h post-infection using the BrightGlo assay according to the manufacturer’s instructions in a Pherastar plate reader. To generate Jurkat cells stably expressing the mouse Na^+^/K^+^ ATPase, *mus musculus* Na^+^/K^+^ transporting alpha 1 polypeptide cDNA (Origene MC204215, Rockville, MD, USA) was cloned into the MLV-based retroviral vector pMIG Zeo [76] (gift of Jeremy Luban, UMASS, USA). Virus was produced in 293T cells as described and used to transduce Jurkat cells, which were selected in the presence of 1μM ouabain for 10 days.

### Primary cells

Peripheral blood mononuclear cells (PBMCs) were isolated from leukapheresis of HIV-1 negative subjects by Ficoll-Paque (Amersham Biosciences) gradient density centrifugation. Memory CD4+ T-cells were sorted from PBMCs by negative selection using magnetic beads (CD4+ T-cell Isolation Kit or Memory CD4+ T-cell Isolation Kit, Miltenyi, Bergisch Gladbach, Germany) with a purity >95%, as determined by staining with CD3-Pacific blue (UCHT1), CD4-Alexa Fluor 700 (RPA-T4), CD45RA-allophycocyanin/Cy7 (HI100) (BD Pharmingen, San Diego, CA), CD8-FITC (BW135/80) (Miltenyi) and FACS analysis [77]. Purified memory CD4+ T-cells were stimulated *via* CD3/CD28 (immobilized CD3 Abs and soluble CD28 Abs, NA/LE, 1 μg/ml, BD Pharmingen, Oxford, UK) and cultured in RPMI1640 media containing 10% FBS, 1% penicillin/streptomycin (PS) for 3 days. Macrophages were isolated by standard Ficoll-Hypaque density centrifugation and incubated at a density of 5x10^4^/well in a volume of 100μL in 96 well plates for 72h in the presence of 20ng/ml of GM-CSF (BD Biosciences) before media change and infection at day 5. PBMCs were transferred into RPMI medium containing 10% FCS and activated with 2μg/mL phytoemagglutinin (PHA) (Sigma Aldrich) for 48h, media was changed and cells were infected and grown for three days in the presence of 10U/mL IL-2 (R&D Systems, Wiesbaden, Germany). Cells were exposed to VSV-G pseudotyped HIV-1 LAIΔenv WT (25 ng CAp24/10^6^ cells) or mutant N74D (50 ng CAp24/10^6^ cells), for 3 hours at 37°C. Unbound virus was removed by extensive washing and cells were cultured in media containing IL-2 (5 ng/ml) in the presence or absence of digoxin (10, 50, 100, 200, and 300 nM) for 40 hours. Cells were harvested and stained with FITC-conjugated CD38 Abs (BD Biosciences), as previously described [78]. A cell viability “Vivid staining” kit (Molecular Probes LIVE/DEAD Fixable Dead Cell Stain Kits, Invitrogen, Thermo-Fisher, Waltham, MA, USA) was used to exclude dead cells from the analysis. The expression of CD38 on viable cells was analyzed using the BD LSRII cytometer (BD). Expression levels of CD154/CD40L were measured as previously described [79] [80]. Briefly, purified memory CD4+ T-cells were stimulated *via* CD3/CD28 and cultured for 3 days. After 24 hours with digoxin treatment, cells were exposed to 2 μM of monensin (Sigma) and 10 μL/wells of FITC-conjugated CD40L Abs (BD Biosciences) for 16 hours at 37°C. Cells were then harvested, stained for cell viability and analyzed by flow cytometry.

### High throughput screening

HTS was performed in 384 well plates (white flat bottom from Greiner) using a Cybio Cybi-Well Vario liquid handling robot with a 384 head plus CyBi-Drop and V&P 384 pin tool to accurately transfer 100 nL of compounds from the master plates. The entire system is enclosed in a special cabinet located in a category 3 laboratory. WT and N74D viruses were pre-mixed at 4°C at a ratio to have approximately 10% of cells infected with each virus. The virus mix was added to the cells in batch, incubating at 4°C with gentle mixing. 5x10^4^ cells/well in 50μl were plated in 384-well plates and 100 nL compound were added to each well (at 1μM final concentration). After 36 hours incubation at 37°C, cells were fixed and analyzed by HTS flow cytometry using a BD LSR II automated to 384-well plates. Data were analyzed using FlowJo software, which also provided information on cell viability. A Z’ score was calculated for each plate. Hit compounds detected in plates with a Z’ score between 0.5 and 1 were considered significant. Hits (h) were defined as follows: *h* ≤ (average % GFP+ cells of control–SD)/2 in the absence of mCherry inhibition.

### RNA sequencing

Three flasks containing Jurkat cells (30 mls at 0.8x10^6^/ml) were independently infected with VSV-G pseudotyped LAIΔenv WT or N74D at an MOI of 0.2 in the presence of DMSO or 400nM digoxin. An aliquot of the cells was analysed by FACS 36 hours post-infection and nucleic acids were extracted from the remaining cells using Qiagen RNAeasy Blood & Tissue kit following the manufacturer’s instructions. 250 ng of total RNA was prepared for sequencing following the Illumina TruSeq mRNA (unstranded) protocol, multiplexed, and sequenced (V2 High Output kit, 43bp PE) on an Illumina Nextseq to yield an average of > 15 million reads per sample. Data was de-multiplexed using bcl2fastq v 2.16. Read QC reports were generated in Illumina Basespace. Fastq files passing read QC were analysed on the UCL Genomics cluster using an in house pipeline (Python/R). Briefly, sequencing reads were aligned to an appropriate reference genome using TopHat v2.0.13 and PCR duplicates removed using Picard tools (v1.100). GTF files describing genes features were obtained from the Ensembl website (http://www.ensembl.org/info/data/ftp/index.html) and read count data for each genomic feature were obtained using the python scripts provided as part of the Deseq2 package. Read count data was subsequently normalized and differential expression computed using Deseq2. Normalized read counts (normalized using the Deseq2 library size procedure) was visualised in Genespring 12.0 (Agilent) and analysed using Ingenuity Pathway Analysis. The set of scripts used is freely available at https://github.com/plagnollab/RNASeq_pipeline.Sequence data can be downloaded from NCBI SRA accession PRJNA321856.

### Integration site analysis

DNA was extracted using Qiagen DNeasy Blood & Tissue kit following the manufacturer’s instructions. Integration sites were identified and quantified in DNA samples as detailed previously [47] using the following HIV-specific PCR forward primers: HIVB3 5’-GCTTGCCTTGAGTGCTTCAAGTAGTGTG-3’, HIVP5B5 5’-AATGATACGGCGACCACCGAGATCTACACGTGCCCGTCTGTTGTGTGACTCTGG-3’ and HIV-specific sequencing primer 5’-ATCCCTCAGACCCTTTTAGTCAGTGTGGAAAATCTC-3’. Prepared sequencing libraries were sequenced on a rapid run of the Illumina HiSeq platform at the Medical Research Council Clinical Sciences Centre Genomics laboratory at Hammersmith Hospital, London, UK. 50 base paired-end reads were aligned to the human genome (hg19) using the eland_pair implementation of CASAVA. *In silico* sites were derived by random sampling of 100,000 sites from the gap-excluded reference of the human genome (hg19). Genomic positions of genes were retrieved from UCSC table browser [81] or published data. Specific gene lists (i.e. genes downregulated by digoxin) were compiled by deriving a non-repetitive list of genes from Ingenuity pathway analysis output and matched against UCSC gene tables to derive genomic coordinates. Integration sites were annotated with these genomic positions using the R Bioconductor package hiAnnotator [82]. Statistical analysis was carried out using R version 2.15.2 (http://www.R-project.org/).

### qPCR

For Taqman qPCR, approximately 1x10^6^ cells were washed twice in PBS and total DNA was extracted with the Qiamp DNA Minikit (Qiagen, Manchester, UK). Quantitative PCR reactions were carried out as previously described [74] using 0.3pmol each primer and 0.15 pmol of the probe in 25 μL volume containing 100-300ng total DNA using an ABI Prism 7000 Sequence Detection System (SDS). For amplification of (-) DNA strand (GFP), primers used were forward: CAACAGCCACAACGTCTATATCAT, reverse ATGTTGTGGCGG ATCTTGAAG and probe 5’-FAM-CCG ACA AGC AGA AGA ACG GCA TCA A-3’TAMRA. For amplification of 2LTR circular DNA, the same conditions were used with primers 2LTRqPCR-F: 5’-AACTAGAGATCCCTCAGACCCTTTT-3’ and 2LTRqPCR-RC: 5’-CTTGTCTTCGTTGGGAGTGAATT-3’ and 2LTR probe 5’-FAM-CTAGAGTTTTCCACACTGAC-0-TAMRA-3’. Standards were prepared by PCR amplification of DNA from acutely infected cells with primers 2LTRF 5’-GCCTCAATAAAGCTTGCCTGG-3’ and 2LTRRC 5’-TCCCAGGCTCAGATCTGGTCTAAC-3’. The amplification product was cloned into TOPO vector, amplified and confirmed by sequencing [16]. Alu-LTR Taqman qPCR was carried out as previously described [16] using primers ALU-forward, AAC TAG GGA ACC CAC TGC TTA AG and LTR1-reverse, TGC TGG GAT TAC AGG CGT GAG (for first round amplification) and ALU-forward AAC TAG GGA ACC CAC TGC TTA AG, LTR2-reverse, TGC TAG AGA TTT TCC ACA CTG ACT, ALU-probe, FAMRA–TAG TGT GTG CCC GTC TGT TGT GTG AC–TAM (for second round Taqman qPCR).

### CD40L assay

Jurkat cells were plated in round-bottom 96 well plates (10^5^/well/100μL) and infected with VSV-G pseudotyped LAI-GFPΔenv in the presence of serial dilutions of anti-hCD40L-Ig antibody (InvivoGen MAbg-h401-3). Cells were analysed by FACS 48h post-infection.

## Supporting information

S1 Fig Linearity of the infection assays. (A) Jurkat cells were infected with different doses of VSV-G pseudotyped HIV-1 vectors WT or N74D and analyzed by flow cytometry 48 hours later. WT vector expressed mCherry (CHE) and the N74D vector expressed GFP. (B) Jurkat cells infected with WT or N74D HIV-1 vectors were exposed to the indicated concentrations of digoxin and analysed by flow cytometry 48 hours later. (C) 1G5 Jurkat reporter cells were infected with HIV-1 NL4.3 WT or N74D and the amount of luciferase assessed at the indicated time points. (D) Primary memory CD4^+^ T cells from 9 donors were infected with HIV-1 LAIΔenv WT or N74D in the absence of digoxin and infection levels (GFP+ cells) measured 48 hours post-infection. Lines connect the same donor. (E) Jurkat cells were infected with different concentrations of HIV-1 LAIΔenv WT or N74D and analysed by flow cytometry 48 hours post-infection. Two separate viral stocks were tested.
(JPG)

S2 Fig Digoxin inhibits HIV-1 gene expression in CD4+ T-cells. (A) Jurkat cells were infected with VSV-G pseudotyped WT HIV-1 LAIΔenv expressing GFP (LAI_GFP_) in the presence of the indicated doses of digoxin and cells were analyzed by flow cytometry 48 hours post-infection. Digoxin inhibited HIV-1 infection with an IC_50_ ≈ 160nM. (B-D) Jurkat cells were infected as above in the presence of digoxin (400 nM), nevirapine (50 nM) or DMSO and DNA was extracted from the cells 24 or 48 hours after infection. The amount of total viral DNA (B), 2LTR circular DNA (C) and integrated viral DNA (D) was quantified by TaqMan qPCR. Mean values ± SD are shown, N = 3. (E-F) Jurkat cells were infected as before and 24h - 36h post-infection they were treated with 400nM digoxin for 24h before analysis by flow cytometry to determine the mean fluorescence intensity (MFI) (E) and the percentage of infected (GFP+) cells (F). (G) Jurkat cells were infected for 24h as described in (B), treated with the indicated doses of digoxin and the amount of HIV-1 *pol* mRNA quantified by RT-qPCR 36h later. Mean values ± SD are shown, N = 3. (H) Jurkat cells infected with LAI_GFP_ with or without 20μM raltegravir (RALT) and the indicated concentrations of digoxin. Cells were analysed by flow cytometry 48h post-infection to measure the percentage of GFP+ cells within the live cell population. Mean values ± SD are shown of an experiment performed in triplicate, which is representative of three independent experiments. (I) Cells infected in parallel were analysed by flow cytometry 48h and 10 days post-infection to confirm the effect of raltegravir.
(JPG)

S3 Fig Diagram showing the experimental design used to perform parallel global RNAseq and integration targeting. Three aliquots of Jurkat cells were independently infected with VSV-G pseudotyped single cycle HIV-1 LAIΔenv WT or N74D mutant in the presence of 400nM digoxin or DMSO. Thirty-six hours post-infection, nucleic acids were extracted and used for RNAseq or integration targeting analyses.
(JPG)

S4 Fig Clustering analysis of RNAseq expressed genes was performed using GeneSpring. Three aliquots of Jurkat cells were independently infected with VSV-G pseudotyped HIV-1 LAIΔenv WT or N74D mutant in the presence of 400nM digoxin or DMSO. Thirty-six hours post-infection, nucleic acids were extracted and used for RNAseq. One sample (DMSO WT 1) did not pass quality control and could not be used for RNAseq.
(JPG)

S5 Fig Summary of integration site analysis. (A) Summary of integration sites in Jurkat cells infected with single cycle, VSV-G pseudotyped HIV-1 LAIΔenv WT or N74D at an MOI of 0.2 in the presence of DMSO or 400nM digoxin. Thirty-six hours post-infection, DNA was extracted, sheared and integration sites quantified using linker-mediated PCR and deep sequencing. 74, N74D virus; WT, wild type virus. Total clones–the total number of unique integration sites. Shear Sites–the total number of proviruses detected across all unique integration sites. Total duplicates–total number of sequencing reads detected across all unique integration sites. (B-C) Plots showing integration within genes for WT and N74D viruses in the presence of DMSO (upper panel) or digoxin (lower panel). Each bar in the bar plots describes the results of an independent experiment. Grey dashed line describes the random expectation (using in silico generated integration site Files). (B) Plots showing integration within genes. (C) Focusing on those integrations within host genes, plots showing proviral orientation relative to the transcriptional start site of cellular genes.
(JPG)

S6 Fig Digoxin down-regulates expression of CD38 in primary memory CD4+ T-cells. (A) IPA diagram highlighting genes down-regulated by digoxin that are part of the CD38 pathway. Continuous lines indicate direct and experimentally validated interactions between genes; dashed lines indicate experimentally validated, indirect interactions. (B-C) Purified memory CD4+ T-cells were stimulated *via* CD3/CD28, cultured for 3 days and exposed to the indicated concentrations of digoxin for 24h. Cells were labeled with anti-CD38 FITC-conjugated antibodies, stained for cell viability and analyzed by flow cytometry. (B) Representative plot showing the percentage of memory CD4+ T-cells expressing CD38 on their surface in the presence of the indicated concentrations of digoxin (y-axis). (C) The percentage of CD38 positive cells and the MFI was quantified by flow cytometry. Bar graphs represent mean ± SD of 3 donors. Friedman test p-values are indicated on the graphs: *, p<0.05; ** p<0.01.
(JPG)

S7 Fig Model for the selective effect of digoxin. WT virus integrates more frequently than N74D virus within or near genes implicated in T-cell activation or metabolism, which are highly expressed in CD4+ T-cells. These genes are susceptible to down regulation by digoxin. Upon digoxin treatment, the chromatin surrounding these genes becomes repressed, and the provirus is also repressed in tandem. Silencing of the N74D virus is less likely due to its different integration targeting.
(JPG)

S1 File Results of the selective high through put screening. Plates’ layout: each well was pre-loaded with the indicated compound at 1 μM. Jurkat cells were exposed to VSV-G pseudotyped HIV-1 vector expressing GFP (WT) or mCherry (N74D) in bulk for 1 hour at 4°C then identical aliquots added onto each well. Thirty-six hours post-infection, cells were analyzed by flow cytometry to measure the percentage of GFP+ or mCherry+ cells. The values corresponding to each compound are indicated and color-coded.
(XLSX)

S2 File RNAseq reads. 250 ng of total RNA was prepared for sequencing following the Illumina TruSeq mRNA (unstranded) protocol, multiplexed, and sequenced (V2 High Output kit, 43bp PE) on an Illumina Nextseq to yield an average of > 15 million reads per sample. Data was de-multiplexed using bcl2fastq v 2.16. Read QC reports were generated in Illumina Basespace. Fastq files passing read QC were analysed on the UCL Genomics cluster using an in house pipeline (Python/R). Sequencing reads were aligned to an appropriate reference genome using TopHat v2.0.13 and PCR duplicates removed using Picard tools (v1.100). Highlighted in green are the genes whose expression was changed >4 fold by digoxin.
(XLSX)

S3 File Ingenuity Pathway Analysis (IPA) of genes up regulated by digoxin. Genes up regulated > 4 fold by digoxin, as determined by RNAseq, were used to identify specific gene pathways by IPA.
(XLS)

S4 File Ingenuity Pathway Analysis (IPA) of genes down regulated by digoxin. Genes down regulated > 4 fold by digoxin, as determined by RNAseq, were used to identify specific gene pathways by IPA.
(XLS)

S5 File List of unique integration sites (UIS). DNA from Jurkat cells, infected with WT or N74D mutant VSV-G pseudotyped HIV-1 LAIΔenv and treated with 400 nM digoxin or DMSO, was extracted using Qiagen DNeasy Blood & Tissue kit following the manufacturer’s instructions. Integration sites were identified and quantified in DNA samples as detailed previously [47] using the following HIV-specific PCR forward primers: HIVB3 5’-GCTTGCCTTGAGTGCTTCAAGTAGTGTG-3’, HIVP5B5 5’-AATGATACGGCGACCACCGAGATCTACACGTGCCCGTCTGTTGTGTGACTCTGG-3’ and HIV-specific sequencing primer 5’-ATCCCTCAGACCCTTTTAGTCAGTGTGGAAAATCTC-3’. Prepared sequencing libraries were sequenced on the Illumina HiSeq platform. 50 base paired-end reads were aligned to the human genome (hg19) using the eland_pair implementation of CASAVA.
(XLSX)

S6 File Up-regulated genes part of the hereditary and developmental disorder networks. These genes were up regulated > 4 fold by digoxin according to the RNAseq and formed a separate IPA network.
(XLS)

S1 Table List of small compounds that induced a differential infection phenotype in the high through put screening. These compounds were not validated further.
(DOCX)
