# Supplementary figures and images for: Digoxin reveals a functional connection between HIV-1 integration preference and T-cell activation
Source: PLoS Pathog. 2017 Jul 20;13(7):e1006460. doi: 10.1371/journal.ppat.1006460 (PMC5519191; doi:10.1371/journal.ppat.1006460)

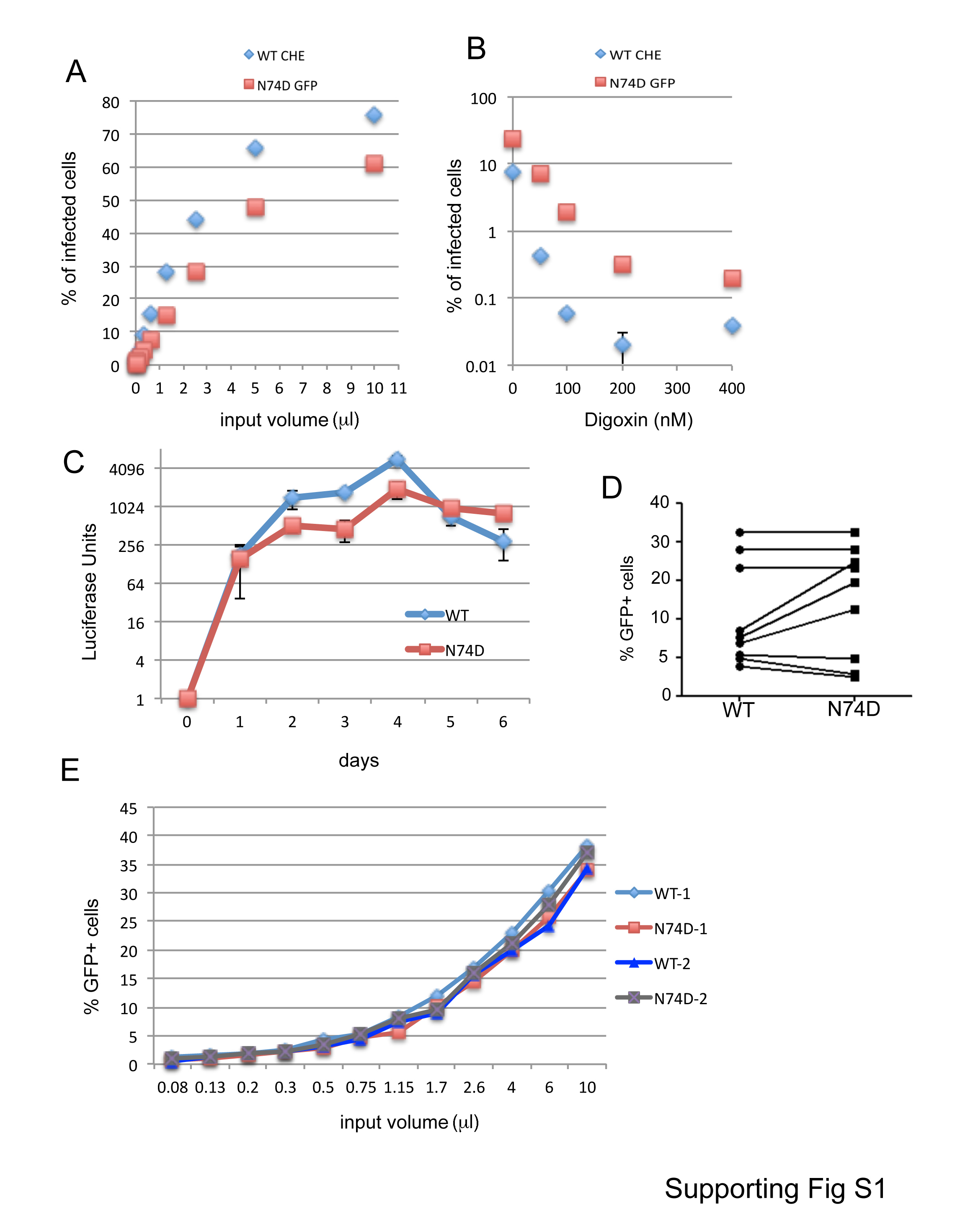

Supplement: S1 Fig — (A) Jurkat cells were infected with different doses of VSV-G pseudotyped HIV-1 vectors WT or N74D and analyzed by flow cytometry 48 hours later. WT vector expressed mCherry (CHE) and the N74D vector expressed GFP. (B) Jurkat cells infected with WT or N74D HIV-1 vectors were exposed to the indicated concentrations of digoxin and analysed by flow cytometry 48 hours later. (C) 1G5 Jurkat reporter cells were infected with HIV-1 NL4.3 WT or N74D and the amount of luciferase assessed at the indicated time points. (D) Primary memory CD4+ T cells from 9 donors were infected with HIV-1 LAIΔenv WT or N74D in the absence of digoxin and infection levels (GFP+ cells) measured 48 hours post-infection. Lines connect the same donor. (E) Jurkat cells were infected with different concentrations of HIV-1 LAIΔenv WT or N74D and analysed by flow cytometry 48 hours post-infection. Two separate viral stocks were tested. (JPG) [file ppat.1006460.s001.jpg]

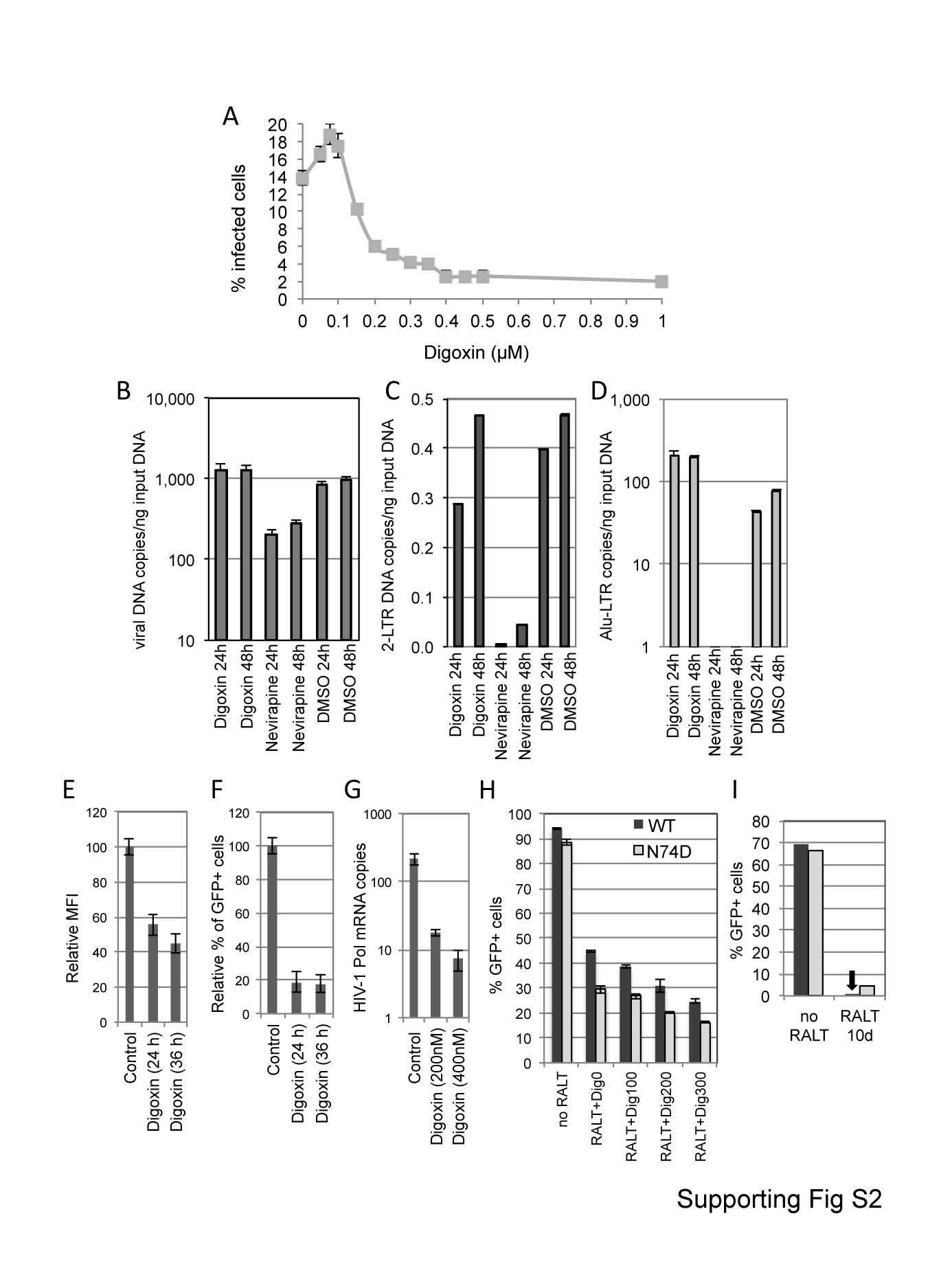

Supplement: S2 Fig — (A) Jurkat cells were infected with VSV-G pseudotyped WT HIV-1 LAIΔenv expressing GFP (LAIGFP) in the presence of the indicated doses of digoxin and cells were analyzed by flow cytometry 48 hours post-infection. Digoxin inhibited HIV-1 infection with an IC50 ≈ 160nM. (B-D) Jurkat cells were infected as above in the presence of digoxin (400 nM), nevirapine (50 nM) or DMSO and DNA was extracted from the cells 24 or 48 hours after infection. The amount of total viral DNA (B), 2LTR circular DNA (C) and integrated viral DNA (D) was quantified by TaqMan qPCR. Mean values ± SD are shown, N = 3. (E-F) Jurkat cells were infected as before and 24h - 36h post-infection they were treated with 400nM digoxin for 24h before analysis by flow cytometry to determine the mean fluorescence intensity (MFI) (E) and the percentage of infected (GFP+) cells (F). (G) Jurkat cells were infected for 24h as described in (B), treated with the indicated doses of digoxin and the amount of HIV-1 pol mRNA quantified by RT-qPCR 36h later. Mean values ± SD are shown, N = 3. (H) Jurkat cells infected with LAIGFP with or without 20μM raltegravir (RALT) and the indicated concentrations of digoxin. Cells were analysed by flow cytometry 48h post-infection to measure the percentage of GFP+ cells within the live cell population. Mean values ± SD are shown of an experiment performed in triplicate, which is representative of three independent experiments. (I) Cells infected in parallel were analysed by flow cytometry 48h and 10 days post-infection to confirm the effect of raltegravir. (JPG) [file ppat.1006460.s002.jpg]

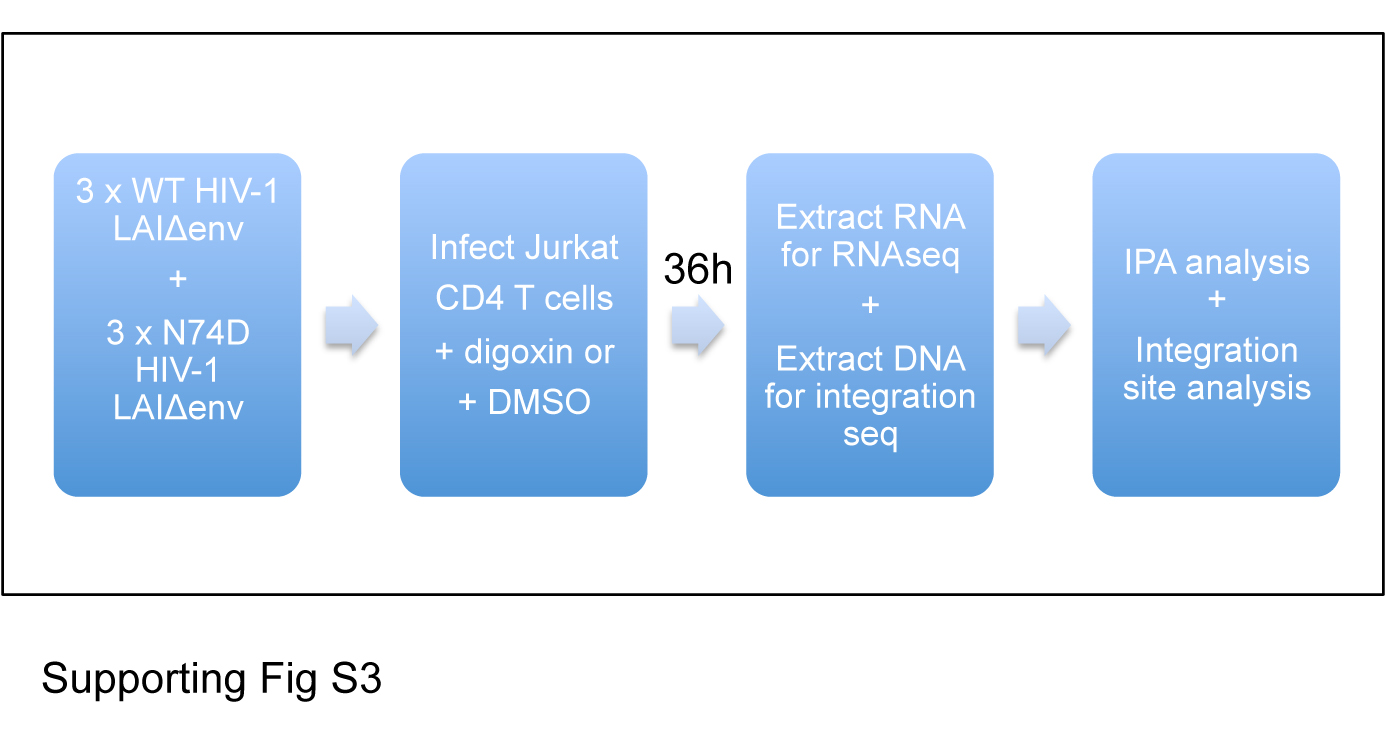

Supplement: S3 Fig — Three aliquots of Jurkat cells were independently infected with VSV-G pseudotyped single cycle HIV-1 LAIΔenv WT or N74D mutant in the presence of 400nM digoxin or DMSO. Thirty-six hours post-infection, nucleic acids were extracted and used for RNAseq or integration targeting analyses. (JPG) [file ppat.1006460.s003.jpg]

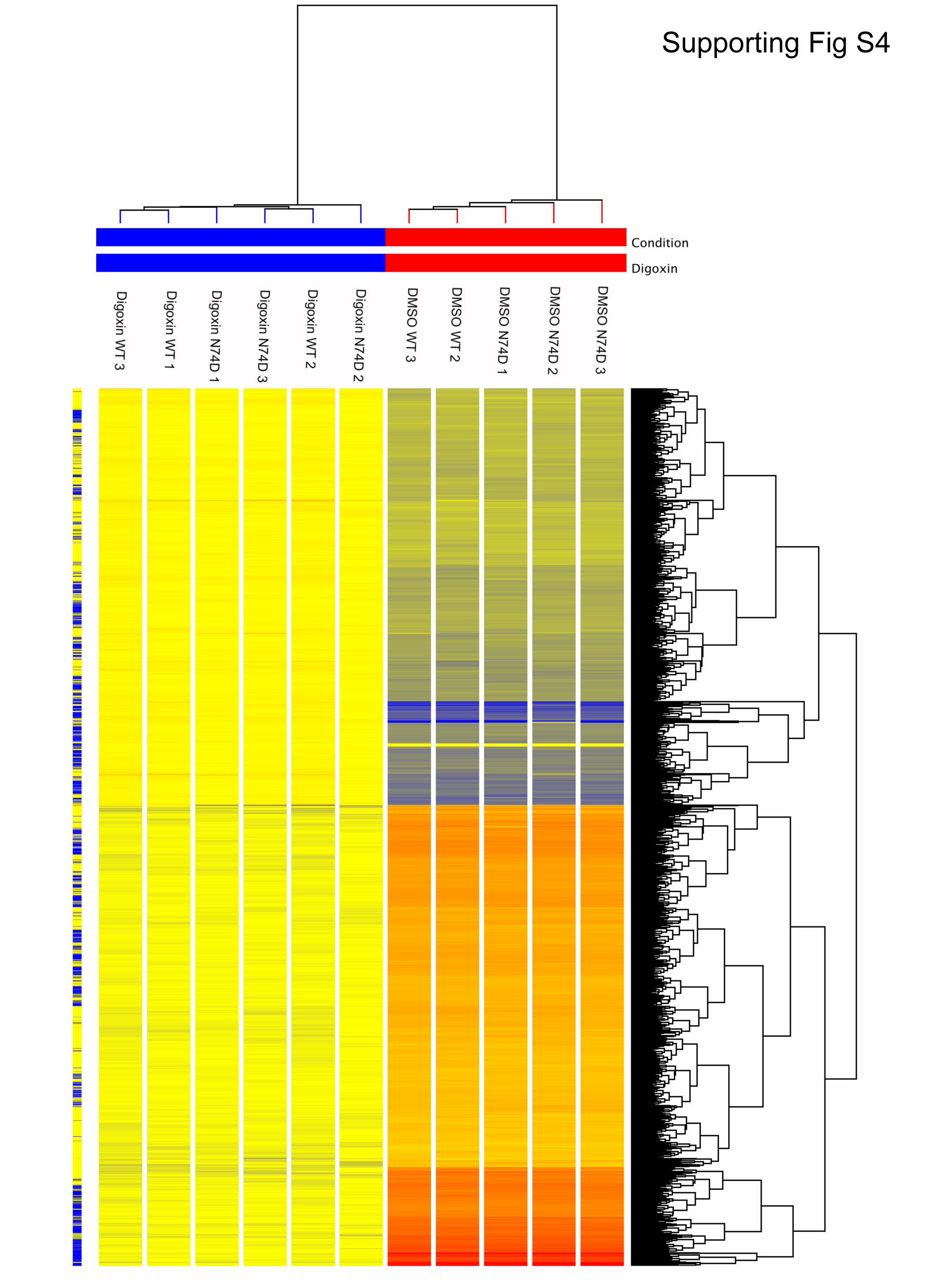

Supplement: S4 Fig — Three aliquots of Jurkat cells were independently infected with VSV-G pseudotyped HIV-1 LAIΔenv WT or N74D mutant in the presence of 400nM digoxin or DMSO. Thirty-six hours post-infection, nucleic acids were extracted and used for RNAseq. One sample (DMSO WT 1) did not pass quality control and could not be used for RNAseq. (JPG) [file ppat.1006460.s004.jpg]

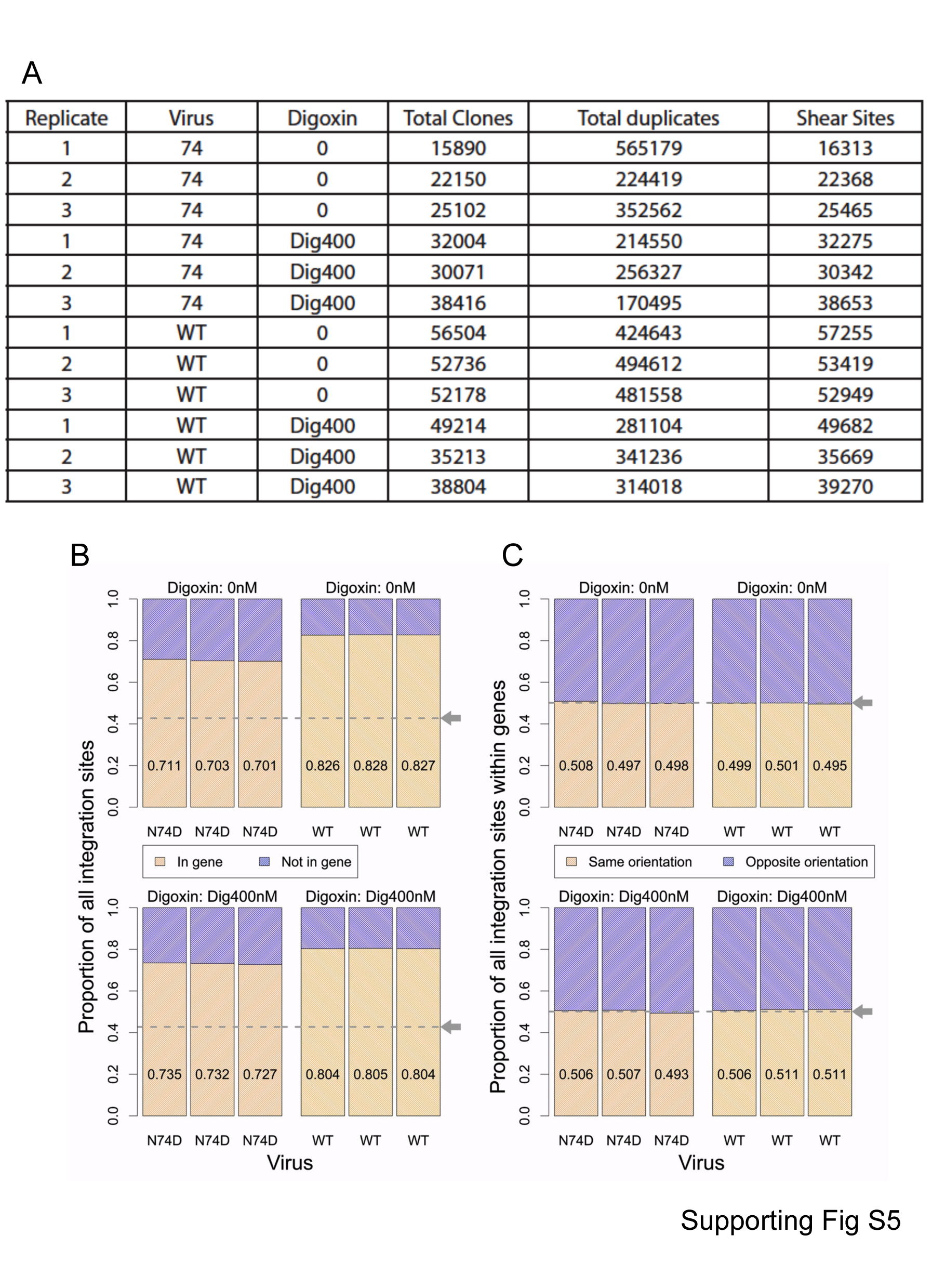

Supplement: S5 Fig — (A) Summary of integration sites in Jurkat cells infected with single cycle, VSV-G pseudotyped HIV-1 LAIΔenv WT or N74D at an MOI of 0.2 in the presence of DMSO or 400nM digoxin. Thirty-six hours post-infection, DNA was extracted, sheared and integration sites quantified using linker-mediated PCR and deep sequencing. 74, N74D virus; WT, wild type virus. Total clones–the total number of unique integration sites. Shear Sites–the total number of proviruses detected across all unique integration sites. Total duplicates–total number of sequencing reads detected across all unique integration sites. (B-C) Plots showing integration within genes for WT and N74D viruses in the presence of DMSO (upper panel) or digoxin (lower panel). Each bar in the bar plots describes the results of an independent experiment. Grey dashed line describes the random expectation (using in silico generated integration site Files). (B) Plots showing integration within genes. (C) Focusing on those integrations within host genes, plots showing proviral orientation relative to the transcriptional start site of cellular genes. (JPG) [file ppat.1006460.s005.jpg]

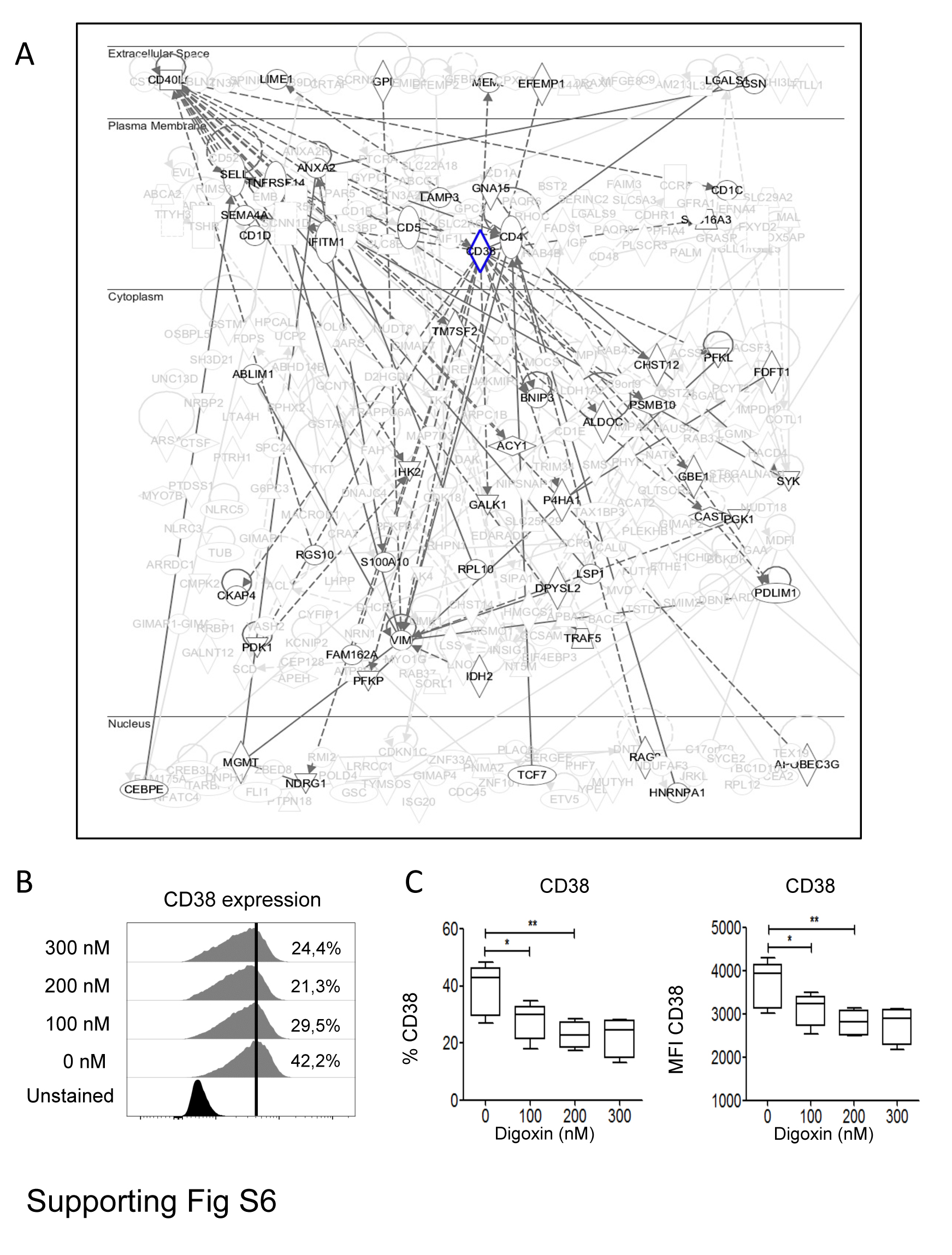

Supplement: S6 Fig — (A) IPA diagram highlighting genes down-regulated by digoxin that are part of the CD38 pathway. Continuous lines indicate direct and experimentally validated interactions between genes; dashed lines indicate experimentally validated, indirect interactions. (B-C) Purified memory CD4+ T-cells were stimulated via CD3/CD28, cultured for 3 days and exposed to the indicated concentrations of digoxin for 24h. Cells were labeled with anti-CD38 FITC-conjugated antibodies, stained for cell viability and analyzed by flow cytometry. (B) Representative plot showing the percentage of memory CD4+ T-cells expressing CD38 on their surface in the presence of the indicated concentrations of digoxin (y-axis). (C) The percentage of CD38 positive cells and the MFI was quantified by flow cytometry. Bar graphs represent mean ± SD of 3 donors. Friedman test p-values are indicated on the graphs: *, p<0.05; ** p<0.01. (JPG) [file ppat.1006460.s006.jpg]

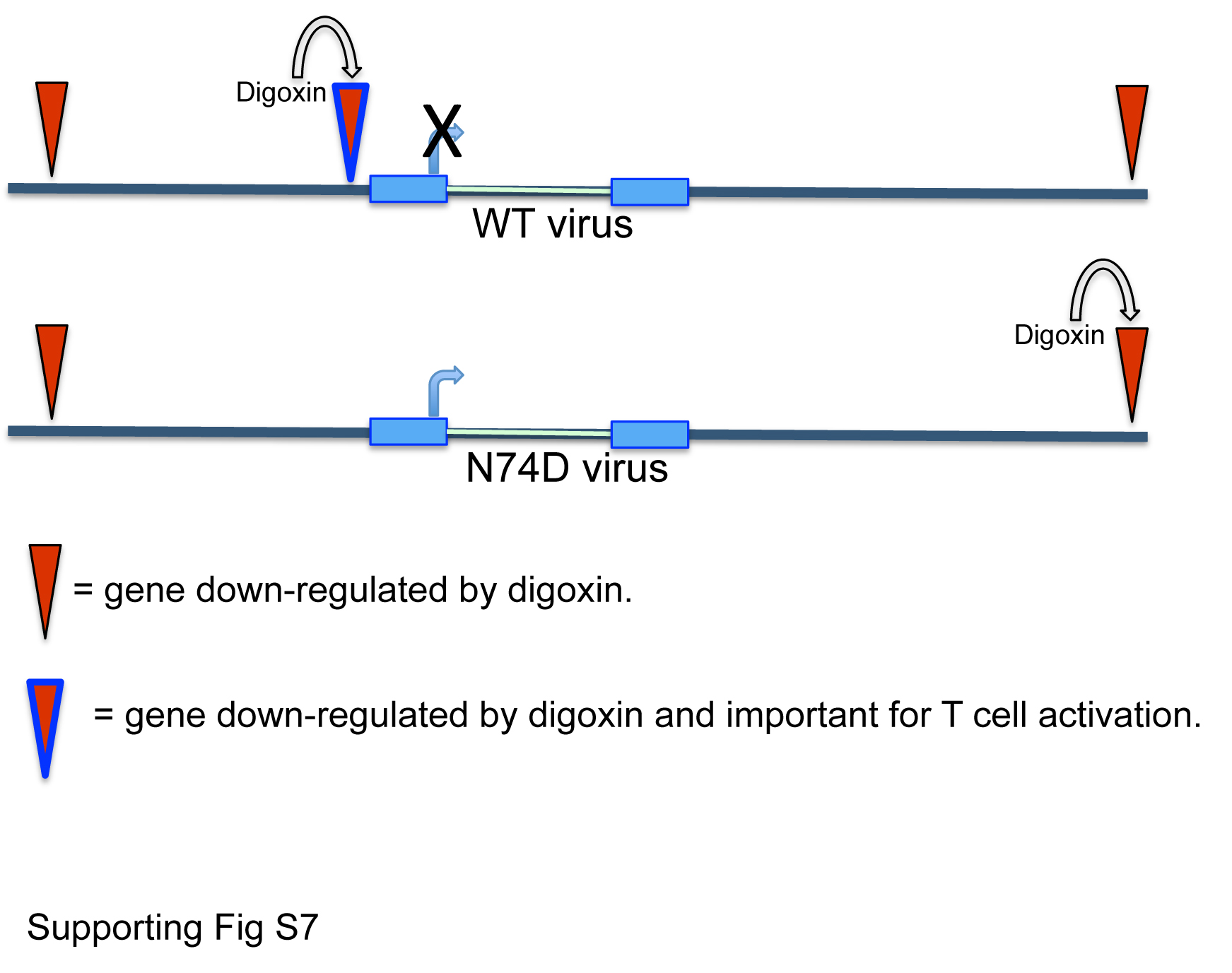

Supplement: S7 Fig — WT virus integrates more frequently than N74D virus within or near genes implicated in T-cell activation or metabolism, which are highly expressed in CD4+ T-cells. These genes are susceptible to down regulation by digoxin. Upon digoxin treatment, the chromatin surrounding these genes becomes repressed, and the provirus is also repressed in tandem. Silencing of the N74D virus is less likely due to its different integration targeting. (JPG) [file ppat.1006460.s007.jpg]
