# Supplementary material for: Digoxin reveals a functional connection between HIV-1 integration preference and T-cell activation
Source: PLoS Pathog. 2017 Jul 20;13(7):e1006460. doi: 10.1371/journal.ppat.1006460 (PMC5519191; doi:10.1371/journal.ppat.1006460)
Supplement: S1 Table — These compounds were not validated further. (DOCX) [file ppat.1006460.s014.docx]

| Compound | Target | Mechanism of action | Use |
| --- | --- | --- | --- |
| Quercitrin | NF-kB | Inhibitor of NF-kB pathway | Anti-inflammatory |
| Fenbufen | cyclooxigenase | Inhibitor of prostaglandins | Anti-inflammatory |
| Hydrocortisone | Glucocorticoid receptor | Agonist | Anti-inflammatory |
| Isoliquiritigenin | Heme oxygenase-1 | Inducer | Anti-inflammatory |
| Sparteine sulfate | Na+ channel | Antagonist | Anti-arrhythmic |
| Lidocaine Hydrochloride | Na+ channel | Antagonist | Local anesthetic |
| Fendiline hydrochloride | Ca++ channels (non selective) | Antagonist | Ca++ channel blocker |
| Digoxin | Na+/K+ ATPase  RORγ/γt | Antagonist | Cardiac glycoside |
| Digitoxin | Na+/K+ ATPase  RORγ/γt | Antagonist | Cardiac glycoside |
| Thiamphenicol | peptidyl transferase (bacteria), DNA synthesis (mammalian) | Inhibitor | Antibiotic |
| Tinidazole | DNA | DNA damage | Anti-infective |
| Acetylcarnitine | Fatty acid transporter | Stimulates use of fatty acids | Dietary supplement |
| Peoniflorin | Androgen receptor | Antagonist |  |
| Beta-propriolactone | Not known |  | Disinfectant |
| Kinetin | Not known | Stimulates cell division | Anti-aging |
| Exalamide | Not known |  | Anti-fungal |
| Silibinin | Not known | Not known | Epato-protective |
| Carbetapentane | Not known | Surfactant | Expectorant |

**S1 Table**. List of selective hits.
